# Porous covalent organic framework liquid for boosting CO_2_ adsorption and catalysis via dynamically expanding effect

**DOI:** 10.1093/nsr/nwaf032

**Published:** 2025-01-24

**Authors:** Zi-Ao Chen, Lei Zou, Rong Cao, Yuan-Biao Huang

**Affiliations:** State Key Laboratory of Structural Chemistry, Fujian Institute of Research on the Structure of Matter, Chinese Academy of Sciences, Fuzhou 350002, China; University of Chinese Academy of Sciences, Beijing 100049, China; State Key Laboratory of Structural Chemistry, Fujian Institute of Research on the Structure of Matter, Chinese Academy of Sciences, Fuzhou 350002, China; Fujian Science & Technology Innovation Laboratory for Optoelectronic Information of China, Fuzhou 350108, China; State Key Laboratory of Structural Chemistry, Fujian Institute of Research on the Structure of Matter, Chinese Academy of Sciences, Fuzhou 350002, China; University of Chinese Academy of Sciences, Beijing 100049, China; Fujian Science & Technology Innovation Laboratory for Optoelectronic Information of China, Fuzhou 350108, China; State Key Laboratory of Structural Chemistry, Fujian Institute of Research on the Structure of Matter, Chinese Academy of Sciences, Fuzhou 350002, China; University of Chinese Academy of Sciences, Beijing 100049, China; Fujian Science & Technology Innovation Laboratory for Optoelectronic Information of China, Fuzhou 350108, China

**Keywords:** covalent organic frameworks, porous liquids, breathing effect, CO_2_ adsorption, cycloaddition reactions

## Abstract

The features of intrinsic porosity and fluidity endow porous liquids (PLs) with unique properties and performance but the preparation of PLs remains challenging due to the difficulty in liquefying and keeping porous features at the same time. Herein, we develop a stepwise surface functionalization and ion exchange strategy to achieve a rare example of flexible covalent organic framework (COF)-based PL (COF-PLs). By coating the outer surface of the judiciously selected three-dimensional flexible COF-301 with an imidazolium salt corona, followed by liquefaction using the anion canopy potassium poly(ethylene glycol) sulfonate (PEGS) via electrostatic interactions, a flexible PL, COF-301-PL, can be obtained. Theoretical calculations and CO_2_ adsorption experiments reveal that the cavities of COF-301-PL undergo dynamic adjustments in response to changes in CO_2_ pressure. The dynamic expansion effect can not only provide additional gas adsorption capacity (7.04 mmol/g at 40 bar) but also facilitate the mass transfer of gas molecules during the catalytic process. Consequently, COF-301-PL exhibits superior catalytic efficiency for the conversion of CO_2_ into cyclic carbonate by a factor of 24 and 17 compared to those of PEGS and COF-301 solid counterpart, respectively. The optimization of substrate adsorption and mass transfer conditions consequently improves the overall efficiency of catalytic reactions. This work offers a new perspective on the preparation of PLs and their great potential application for gas adsorption and catalysis.

## Teaser

This work develops a novel approach to synthesize a porous liquid based on the flexible covalent organic framework (COF), which exhibits exceptional performance in CO_2_ capture and catalysis.

## INTRODUCTION

Porous solid materials have great potential in applications of gas separation [1,2] and catalysis [3,4] because of their precise pore structure [5,6], large surface area [7,8], and tunable adsorption units and active sites [9,10]. However, their suboptimal heat and matter transfer efficiency along with mechanical fatigue features have limited these porous solids for practical applications [11,12]. Particularly, the pores of the solid materials are usually occupied by solvents in the gas-liquid-gas reaction systems [13,14]. Moreover, the gas solubility is usually poor in traditional liquids. Therefore, only a small amount of reactive gas can access and contact with active sites through the pores and thus their catalytic conversion rates still need to be improved to meet practical requirements. In contrast, the recently developed porous liquids (PLs) that combined the advantages of porous solids (with permanent, rigid and well-defined pores) and liquids (fluidity, fast heat and mass transfer) could provide an alternative promising strategy to enhance gas adsorption [15–17] and catalytic [18–20] performances in liquid. This is due to higher gas capacities of PLs in the liquid state, along with the physical adsorption interactions with gases, which contribute to rapid gas exchange kinetics in comparison with solid catalysts and solvents [21–23]. The enhancement of gas concentration in catalysis processes through PLs offers an efficient catalytic pathway, as the mass transport limitations imposed by the diffusion of gaseous substrates to the catalyst surface are minimized or entirely circumvented. This makes PLs more suitable for gas-liquid interface multiphase catalytic reactions.

Although the concept of PLs had been proposed in 2007 [24], the first example of PLs were not synthesized by James *et al.* till 2015 due to the difficulty in preparation caused by the contradiction between porosity and fluidity [25,26]. Although several preparation approaches including coordination self-assembly and surface engineering strategies have been developed to create PLs, only a small portion of PLs based on hosts such as porous zeolites [27,28], porous silica [29,30], porous carbon [31,32], metal-organic cages (MOCs) [33,34] and metal-organic frameworks (MOFs) [35–38] have been reported. However, most of the reported PLs have rigid pores that lack dynamic responsiveness, resulting in their adsorption capacity being limited by pore volume and surface area [37,39], without the ability to increase effective adsorption sites or expand pore volume through structural responsiveness [40]. As is well known, compared with the rigid host in the physisorption process, the adaptive interactions between the flexible host and gas can inherently drive the gas adsorption-desorption dynamics and regulate the mass transfer process, which can enhance the adsorption capacity at a broader working pressure range [41]. Therefore, it is highly desirable to design and synthesize new stable PL materials based on flexible hosts with higher performance over a wider working pressure range in order to promote catalysis efficiency.

Compared to the aforementioned porous hosts, the porous crystalline covalent organic frameworks (COFs) constructed by robust covalent bonds have shown strong chemical stability in various harsh environments [42]. More importantly, the dynamic flexible building blocks, displacement between interwoven frameworks, and tunable configuration of linkages endow COFs with strong flexibility, enabling responsive changes in pore cavities under varying gas pressures [43,44]. As a result, COFs-based PLs can adapt to a wide range of gas pressures, effectively capturing gas molecules and enhancing mass transfer to improve catalytic efficiency. However, to date, there has been no report on using COFs to construct PLs for heterogeneous catalytic reactions. This challenge arises from the large apertures of most COFs, which allow solvent molecules or liquefaction groups to fill the pores, leading to porosity loss. Moreover, liquefying two-dimensional (2D) or three-dimensional (3D) COFs with large particle sizes remains particularly challenging. It is also essential to control the interaction between the solid matrix and liquefaction groups, and prevent aggregation and sedimentation, which would compromise liquidity. To preserve the permanent porosity of flexible COFs, preventing liquefaction agents from penetrating the pores is critical.

**Scheme 1.**
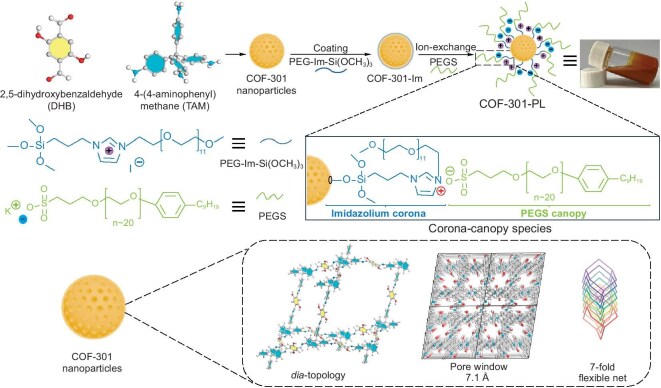
Schematic of the synthesis of the stable porous COF liquid COF-301-PL through the strategy of constructing corona-canopy electrostatic interaction interfaces.

Herein, for the first time, we report a flexible COF-based PL (COF-301-PL) for CO_2_ catalytic conversion via surface functionalization of a 3D microporous COF-301 with positively charged polyethylene glycol and organosilane co-functionalized imidazolium salt (PEG-Im-Si(OCH_3_)_3_), which was further liquefied by anchoring a negatively charged poly(ethylene glycol)-tailed sulfonate (PEGS) canopy with an ion-exchange strategy (Scheme 1). COF-301 was selected because of its ultra-micropore window (7.1 Å) and 7-fold interpenetrated flexible structure, which prevents the penetration of PEG-Im-Si(OCH_3_)_3_ coronas (18.8 Å) and PEGS canopies (50.5 Å), thus maintaining PL porosity. Molecular dynamics (MD) simulations show that strong electrostatic interactions between the cationic corona-canopy species and PEGS anions are key to the dispersion and stability of the porous host. As a result, COF-301-PL demonstrates excellent fluidity and porosity at room temperature. The intrinsic flexibility of COF-301, derived from its distorted tetrahedral building blocks and interpenetrating networks, enables dynamic pore expansion upon CO_2_ filling. CO_2_ adsorption simulations and high-pressure measurements reveal that increasing CO_2_ pressure causes the expansion of the tetrahedral building blocks and displacement of interpenetrated networks, resulting in dynamic cavity expansion. Rietveld refinement of the cell parameters shows that the fully expanded COF-301 framework exhibits a 48.8% increase in unit cell volume and 3.0 times increase in pore volume. Consequently, the theoretical CO_2_ adsorption capacity of the fully expanded COF-301 is 2.9 times that of the pre-expanded state. CO_2_ adsorption experiments on COF-301-PL exhibit similar behavior to COF-301, but with enhanced capacity due to dynamic pore expansion, accelerating the mass transfer rate during catalytic processes. As a CO_2_ reservoir for catalytic reactions, COF-301-PL enables efficient cycloaddition reactions between CO_2_ and epoxides to generate cyclic carbonates. Notably, the catalytic conversion rate of COF-301-PL was 24 and 17 times higher than that of PEGS and COF-301 under the same conditions, respectively.

## RESULTS AND DISCUSSION

### Synthesis and characterization of COF-301 and COF-301-Im nanoparticles

**Figure 1.**
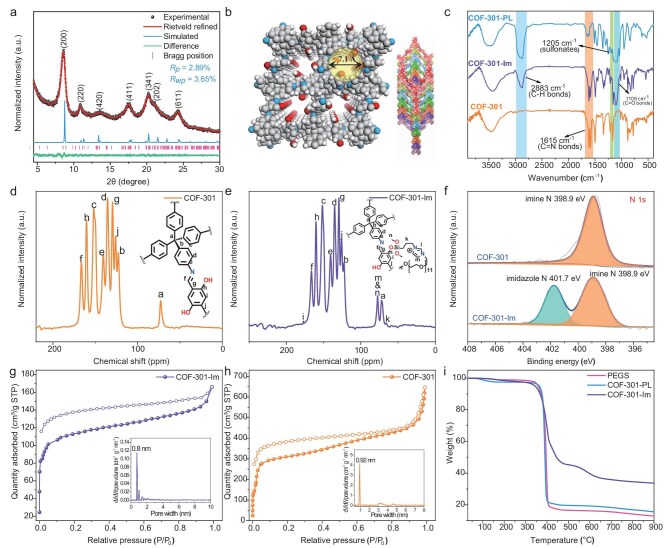
(a) The PXRD pattern and Rietveld refinement of COF-301 framework. (b) Schematic illustration of the flexible *dia*-topology network and the 7-fold interpenetrated structure of COF-301. (c) FT-IR spectra of COF-301, COF-301-Im and COF-301-PL. Soild-state^13^C CP-MAS spectra of COF-301 (d) and COF-301-Im (e). (f) High-resolution N 1s XPS spectra of COF-301 and COF-301-Im. (g) N_2_ sorption isotherms of the COF-301 and (h) COF-301-Im. (i) The TGA curves of PEGS, COF-301-Im and COF-301-PL.

The judicious choice of the typical COF-301 as the porous host is based on its flexible framework with ultra-small aperture, and hydroxyl-rich surfaces (Scheme 1), which can provide anchoring sites to be modified and grafted with the corona layer. The microporous 3D COF-301 nanoparticles were synthesized by the reaction of the diamond-type linker tetrakis(4-aminophenyl) methane (TAM) and 2,5-dihydroxy-1,4-benzodialdehyde (DHB) (details in Section S2). The successful synthesis of COF-301 was confirmed by the appearance of a peak for the C=N bond in the Fourier Transform Infrared (FT-IR) spectroscopy (1622 cm^−1^, Fig. 1d) and ^13^C cross-polarization/magic angle spinning nuclear magnetic resonance (CP/MAS NMR) spectroscopy (155 ppm, Fig. 1e). Transmission electron microscopy (HR-TEM) images reveal that the obtained COF-301 nanoparticles have an average size of ∼50 nm (Fig. S5). Powder X-ray diffraction (PXRD, Fig. 1a) analysis in combination with the Rietveld method demonstrated that COF-301 exhibits a ***dia*** topology with a 7-fold interpenetrating framework, with reasonable factors of *R**_wp_*** = 3.65% and *R**_p_*** = 2.89%. Due to the 7-fold interpenetrating framework, COF-301 has a micropore window of ∼7.1 Å with the same size of aperture (Fig. 1b), which can effectively prevent penetration of the self-filling out-layer corona and canopy species. Meanwhile, the tetrahedral configuration of the TAM building blocks provides substantial rotational freedom to the benzene rings. The C-C single bond between the benzene ring and the central carbon atom allows for bending or stretching within a certain range, enabling the molecule to deform more easily under external forces. Moreover, the different networks within the interpenetrated structure can influence and adjust each other, and the sliding capability between each singly interpenetrated framework further enhances the flexibility and deformability of the COF-301 host under external forces, enabling the construction of PLs responsive to different gas pressures or molecules [45]. Nitrogen adsorption isotherms measured at 77 K (Fig. 1h) reveal a sharp adsorption increase in the pressure range of 0–0.1 (*P*/*P*_0_), displaying typical Type I adsorption isotherm characteristics with a microporous structure feature. The pore size distribution plots based on the quenched-solid density functional theory (QSDFT) verified that the dominant pore size for COF-301 is situated around 0.78 nm, which is in excellent agreement with the simulation result. Moreover, COF-301 has an impressive specific surface area of up to 1004 m^2^/g, and under 273 K and 1.0 atm conditions, CO_2_ capacity reached 49.5 cm³/g (Fig. S10).

The corona was then grafted to the COF-301 by the reaction between its surface-bound hydroxyl groups with polyethylene glycol and organosilane co-functionalized imidazolium (PEG-Im-Si(OCH_3_)_3_) molecules (Figs S1–S4) to obtain the COF-301-Im (Scheme 1). The PEG-Im-Si(OCH_3_)_3_ with a large size of 18.8 Å, (Fig. S6) was employed as a cationic corona layer to coat on the exterior surface of COF-301, which would be favorable in the formation of stable PLs via electrostatic interactions with the negative PEGS canopy. Furthermore, it can prevent the PEGS canopy species from penetrating the pore cavities during subsequent liquefaction processes. The cationic corona layer did not obviously affect the pore structure of COF-301-Im and its porosity was still well maintained, as evidenced by the high BET specific surface area of 442 m²/g, as shown in Fig. 1g. The FT-IR spectroscopy and ^13^C CP-MAS spectrum confirmed the successful coating of COF-301 with PEG-Im-Si(OCH_3_)_3_. As shown in Fig. 1c, compared to the FT-IR spectroscopy of the parent COF-301 sample, additional absorption bands around 1050 cm^−1^ to 1150 cm^−1^ are clearly observed for COF-301-Im, which are attributed to the stretching vibrations of the polyethylene glycol (PEG) chain moiety (–O–CH_2_–CH_2_–O–) and the imidazolium aromatic ring of PEG-Im-Si(OCH_3_)_3_, respectively. Additionally, the new peak at 2883 cm^−1^, corresponding to the stretching vibration of aliphatic –C–H bonds, indicates the successful evolution of the silane with a –(CH_2_)_3_– group onto the COF-301 framework. In the ^13^C CP-MAS spectrum of COF-301-Im, new chemical shift signals are observed at 62.4 ppm, 77.6 ppm and 167.8 ppm. These peaks are ascribed to the carbon atoms of the methylene, –O–CH_2_–CH_2_-O groups and the imidazolium ring from PEG-Im-Si(OCH_3_)_3_ moiety (Fig. 1d and e). The above results suggested that the corona PEG-Im-Si(OCH_3_)_3_ is chemically bonded to the surface of COF-301, rather than being merely a physical coating. X-ray photoelectron spectroscopy (XPS) analysis of COF-301-Im revealed the presence of C, N, O, Si and I elements (Fig. S13), suggesting that the corona PEG-Im-Si(OCH_3_)_3_ was grafted to the COF-301. Moreover, the XPS spectrum of COF-301 in Fig. 1f shows that only the N 1 s peak at 398.9 eV for imine bond is observed. While for COF-301-Im, a distinct additional N 1 s peak at 401.8 eV appears, which is assigned to the quaternary nitrogen species of the imidazolium groups. Meanwhile, a peak at 619.2 eV for COF-301-Im is observed and can be ascribed to the I 3D orbital, confirming the presence of free I^−^ ions (Fig. S13). The presence of I^−^ anions in COF-301-Im facilitates contact of the cationic corona with the anionic canopy PEGS molecules via an ion exchange method in the subsequent steps.

### Synthesis and characterization of COF-301-PL

The COF-301-PL was obtained by substituting the exterior surface counter-anion I^−^ of COF-301-Im with a negative PEGS canopy (details in SI Section S2). The elemental mapping images of COF-301-PL show that the Si species are mainly located at the edges of the host COF particles (Fig. S5d), indicating the surface of COF-301 was coated by the PEG-Im-Si(OCH_3_)_3_ corona layer. Meanwhile, only a little of the counter-anion I^−^ element is observed at the periphery of the porous host of COF-301-PL, which indicates that most of the I^−^ ions have been exchanged by the PEGS canopies (Fig. S5g). Compared with the FT-IR spectrum COF-301-Im, additional distinctive bands of sulfonates (1205 cm⁻¹) and ethers (1109 cm⁻¹) are clearly observed for COF-301-PL (Fig. 1c). Moreover, a significant increase in peak intensity at 2883 cm⁻¹, corresponding to the aliphatic C-H stretching mode, appeared. These results suggest that the negative PEGS shields have replaced the I^−^ anions of COF-301-Im. TGA demonstrated that the COF-301-PL is thermally stable up to 380°C (Fig. 1i).

**Figure 2.**
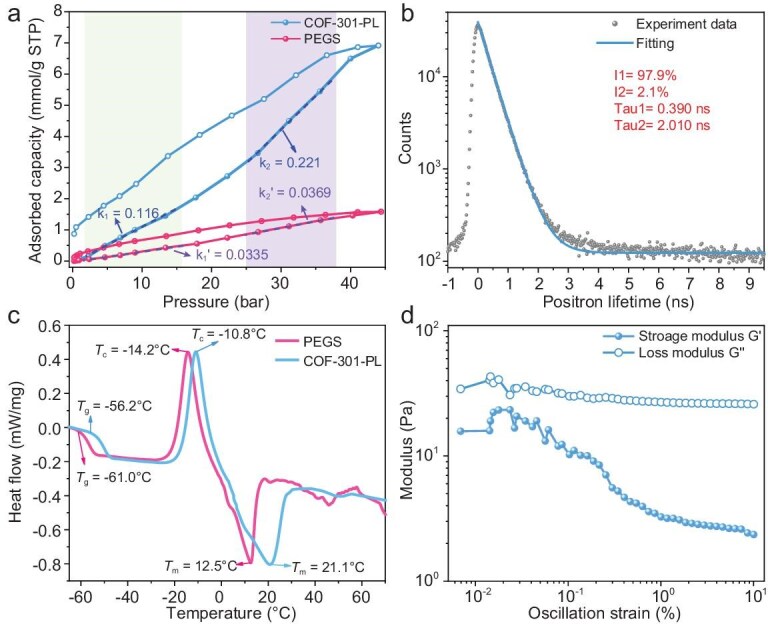
(a) The high-pressure CO_2_ adsorption isotherms of pure PEGS and COF-301-PL at 298 K (0–43 bar). (b) The positron lifetime traces of COF-301-PL collected at 298 K. (c) The DSC curves of pure PEGS and COF-301-PL. (d) Oscillation-dependent modulus plots of COF-301-PL (filled circles for storage modulus G' and open circles for loss modulus G'').

To confirm the permanent porosity of COF-301-PL, geometry optimization calculations of PEGS and PEG-Im-Si(OCH_3_)_3_ moieties were performed using the Gaussian 16 program. As shown in Fig. S6, the dimensions of the PEGS molecule (50.5 Å × 21.5 Å × 11.5 Å) and PEG-Im-Si(OCH_3_)_3_ molecule (18.8 Å × 14.5 Å × 8.4 Å) are significantly larger than the pore size of COF-301 (7.1 Å), fundamentally preventing them from penetrating the cavities of the COF host. In contrast, the CO_2_ molecule with a small size of 5.6 Å × 3.4 Å × 3.2 Å can penetrate the corona-canopy interface of the COF host, and fill the internal cavities of COF-301-PL. Thus, COF-301-PL exhibits a remarkable adsorption capacity for CO_2_ (7.04 mmol/g), with an adsorption amount 4.5 times greater than non-porous PEGS (1.55 mmol/g) at 43 bar and 298 K (Fig. 2a). It thus suggested that the COF-301-PL has an intrinsic porosity and additional free volume for storing CO_2_ molecules.

Additionally, positron annihilation lifetime spectroscopy (PALS) was used to further determine the porosity in COF-301-PL [30]. Figure 2b shows the positron lifetime spectrum through COF-301-PL and the corresponding fitting curve. The ortho-positronium (*o-Ps*) lifetime and intensity are usually related to the size and concentration of open volumes in the material, respectively. The *o-Ps* lifetime of 2.010 ns corresponds to an average spherical continuous free volume radius of 0.51 nm, which matches well with the pore size of the COF-301 host (the pore window radius of COF-301 is 0.36 nm and the channel radius is 0.47 nm). The PALS results indicate that the pore characteristics of COF-301 are largely retained in COF-301-PL. Consequently, COF-301-PL also inherits the intrinsic flexibility and gas adsorption capacity of the COF framework.

Differential scanning calorimetry (DSC) and oscillatory strain modulus analysis were used to investigate the phase transition behavior and rheological properties of COF-301-PL. As shown in Fig. 2c, a second-order transition corresponding to the glass transition temperature (*T_g_*) was observed at −56.2°C. A distinct exothermic peak at −10.8°C was attributed to the crystallization temperature (*T_c_*). With a further temperature increase, COF-301-PL exhibited a significant first-order transition at +21.1°C, corresponding to the melting temperature (*T_m_*), which inicates the melting of COF-301-PL upon heating. Therefore, at room temperature (25°C), COF-301-PL displays intrinsic liquid characteristics (Scheme 1). Compared to COF-301-PL, the *T_g_*, *T_c_*, and *T_m_* of PEGS are −61.0, −14.2 and +12.5°C, respectively, all lower than those of COF-301-PL. This phenomenon is attributed to the electrostatic attraction between the cationic corona and anionic canopy in COF-301-PL, which induces the formation of a short-range ordered structure at the interface. Consequently, COF-301-PL requires higher energy to overcome this potential barrier, providing additional evidence of strong electrostatic interaction at the interface. Oscillatory strain experiments further quantified the fluidity of COF-301-PL at room temperature. Under shear stress, the loss modulus (G'') is used to describe the damping capacity of the material, while the storage modulus (G') indicates the density of elastic potential energy stored in the material; a higher storage modulus indicates greater rigidity. Accordingly, the loss factor G''/G' is used to define the viscoelastic properties of the material [14,46]. When the loss factor is >1, the material is considered to be viscous, with the shear stress reflecting the internal friction within the fluid. As shown in Fig. 2d, the G'' consistently exceeds G' across the entire range of oscillatory stress, indicating that the loss factor is always larger than 1, thereby revealing the flow behavior of COF-301-PL. These findings confirm the intrinsic liquid nature of COF-301-PL at room temperature.

The strong Coulomb interactions between the cationic corona of the COF-301-Im framework and the anionic PEGS canopy plays an important role in liquefying the COF host and forming stable COF-301-PL, thereby preventing phase separation between the porous host and the PEGS liquefier. Theoretically, the absolute value of the ζ potential is positively correlated with the stability of colloidal systems. Due to van der Waals interactions, dispersions with low ζ potential will ultimately lead to micelle aggregation and precipitation. When the absolute value of the ζ potential exceeds 30 mV, the micelles are considered to have stable dispersion [15]. Compared to the neutral COF-301 framework (−3.31 mV) and the physical mixture of the COF-301 framework with PEGS (−10.32 mV), the COF-301-PL shows a significantly higher ζ potential (+37.29 mV) due to the replacement of the characteristic adsorbed species I^−^ in the inner Helmholtz layer on the outer surface by PEGS anions (as shown in Fig. S14). It demonstrates that the significant Coulomb interactions between the porous host surface and PEGS make COF-301-PL a stable liquid system. In contrast, the physical mixture of COF-301-Im and PEGS without I^−^ exchange exhibits a lower ζ potential (+14.25 mV). Such weak interaction between the porous host and PEGS prevents the formation of a stable PL and ultimately leads to phase separation.

**Figure 3.**
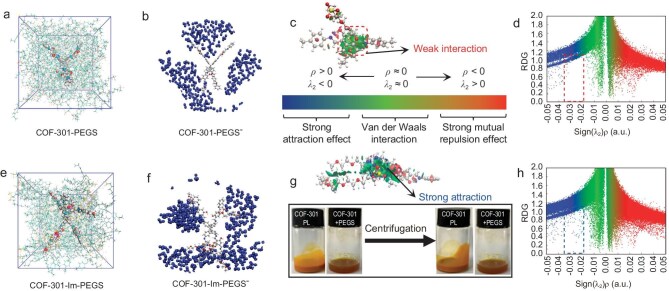
MD snapshots displaying COF-301 (a) and COF-301-Im (e) dispersed in PEGS solvent, respectively. The center-of-mass spatial distribution function (SDF) of PEGS cations (PEGS^−^, blue) around the COF-301 (b) and COF-301-Im (f), respectively. Schematics of the non-covalent interactions (NCIs) between COF-301 with PEGS (c), and COF-301-Im with PEGS (g), respectively. The inset shows photographs of the physical mixture of COF-301 + PEGS and COF-301-PL after centrifugation at 5000 r/min for 5 minutes. The reduced density gradient (RDG) isosurface in the spatial regions of COF-301 (d) and COF-301-Im (h) complexes with PEGS.

To visually demonstrate the stability of COF-301-PL, it was subjected to high-speed centrifugation at 5000 r/min for 5 minutes. Even after this process, no significant phase separation was observed in COF-301-PL, whereas a clear solid-liquid interface was evident in the suspension formed by directly mixing COF-301 with PGES (Fig. 3g). Molecular dynamics (MD) simulations can provide important insights into the molecular mechanisms underlying the sustained stability of COF-301-PL [47]. In this study, we utilized MD simulations and quantum mechanical calculations to elucidate the interaction of COFs with the PEGS anion. As shown in Figs 3e and f, the spatial distribution function (SDF) indicates that the PEGS anions significantly aggregate and enclose around the imidazolium salt side chains of the COF-301-Im fragment. It suggests that the electrostatic interaction between the PEGS anion and positively charged imidazolium salt facilitates their tight association, forming a stable corona-canopy electric double layer (Fig. S8). According to the general paradigm of PEGS molecules, further enhancing host-guest interactions ultimately yields a stable PL. Conversely, the final MD snapshots and SDF diagrams of COF-301 and PEGS (Fig. 3a and b) demonstrated that they cannot bind tightly, and there is almost no significant wrapping or aggregation of PEGS anions around the framework. Consequently, COF-301 and PEGS cannot form stable micelles, and Brownian motion causes continuous collisions between porous particles, leading to coagulation and eventually the sedimentation of the COF dispersed phase. The radial distribution function (RDF) quantitatively illustrates the distribution probability of PEGS anions at various distances from the given framework. For the COF-301-Im fragment, the distribution probability of PEGS near the 0.5 nm shell on the outer surface of the COF is significantly higher than that of the COF-301 framework, clearly indicating that PEGS⁻ forms a tighter interaction with the COF-301-Im nanoparticles. This more comprehensive encapsulation structure further facilitates the liquefaction of the COF solid and enhances the long-term stability of the resulting liquid. Additionally, the range of electrostatic attraction aligns with this distance, further corroborating the existence of the corona-canopy bilayer (Fig. S9). To further understand the interactions between the cationic corona and PEGS in COF-301-PL, we investigated the reduced density gradient (RDG), which visualizes non-covalent interactions in real space [48]. The relationship between the RDG and the corrected electron density (ρ_(**r**)_) is influenced by the sign of the second eigenvalue (λ₂) of the electron density Hessian (Fig. 3c) [49]. In the RDG plot, the additional spike observed around λ₂ = −0.03 a.u. for PEGS and COF-301-Im fragments confirms the presence of additional attractive interactions beyond π-π stacking (Fig. 3d and h). Based on this, as shown in the non-covalent interaction (NCI) plot in Fig. 3g, a significant attractive interface (blue part) can be observed between the imidazolium salts in the COF-301-Im fragment and the PEGS anions, attributed to electrostatic attractions. In contrast, only weak π-π stacking interactions (green part) are present between COF-301 and PEGS (Fig. 3g). The strong electrostatic attraction between the cationic corona of COF-301-Im and the anionic corona of PEGS significantly enhances their interaction and leads to the formation of a stable liquid. These findings underscore the importance of the ionic exchange strategy and the electrostatic interaction interface between corona species in preparing stable porous COF liquids. Collectively, these results demonstrate that COF-301 has been successfully converted into a liquid material while preventing the infiltration of the bulky solvent PEGS into the pores, thus preserving the inherent porosity of the COF-301 framework.

### The CO_2_ adsorption behaviour and catalytic performance of COF-301-PL

Integrating inherent porosity into the liquid phase can provide additional opportunities for specific applications. For example, in traditional gas-liquid-solid three-phase reactions, the low solubility of gas molecules in the solution limits mass transfer efficiency. Additionally, the complex and heterogeneous catalytic microenvironment at the three-phase interface leads to inevitable competition between gas substrates and solvents for catalytic sites. When gas molecules are adsorbed into the permanent pores of the fluidic material, the mass transfer efficiency during phase transitions can be significantly improved. Given the high CO_2_ adsorption capacity of COF-301-PL, using PLs as micro gas reservoirs in multiphase catalytic reactions provides the necessary conditions to overcome mass transfer limitations due to poor gas solubility. Moreover, the intrinsic flexibility of the COF-301 framework endows COF-301-PL with the ability to dynamically adjust in response to external gas pressure changes. This adjustment not only increases gas adsorption but also optimizes the adsorption and mass transfer conditions of gas substrates within the catalytic system. The responsive pores further facilitate rapid gas exchange dynamics, thereby enhancing the overall efficiency of the catalytic reaction. Coupled with the inherent stability of COF-301-PL, it can avoid permanent pore damage and material fatigue through dynamic structural adjustments during prolonged catalytic reactions, thereby improving the durability and sustained stability of the catalytic system.

**Figure 4.**
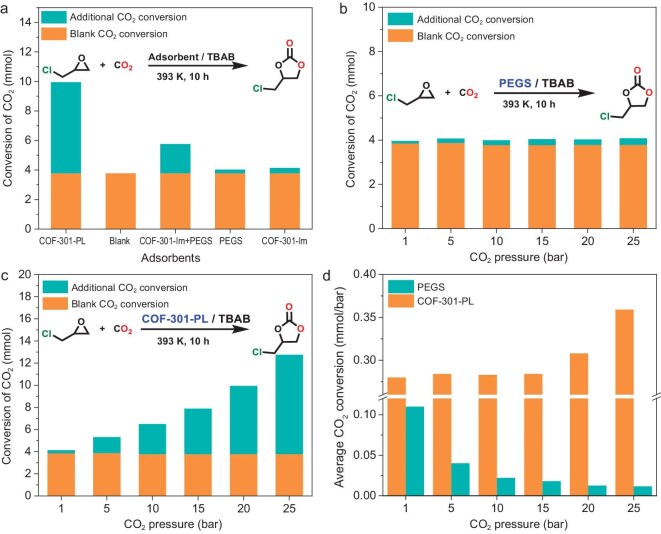
(a) The experimental results of CO_2_ catalytic conversion with different adsorbents. The CO_2_ catalytic conversion of PEGS (b) and COF-301-PL (c) under different gas pressures. (d) The average CO_2_ catalytic conversion increment per 1 bar pressure for PEGS and COF-301-PL under different pressure conditions.

To validate the above concept, COF-301-PL was first applied in the cycloaddition reaction of CO_2_ and epichlorohydrin under atmosphere pressure conditions. Initially, COF-301-PL was fully saturated with CO_2_ at a pressure of 20 bar in a high-pressure reactor. The pressure was then rapidly reduced to atmospheric pressure, and the reactor was sealed immediately. Subsequently, the high-pressure reactor was placed in a liquid nitrogen environment to immobilize the adsorbed CO_2_. Finally, at −196°C, the reactor was opened, and 20 mmol of epichlorohydrin and the catalyst tetrabutylammonium bromide (TBAB) were introduced to conduct the cycloaddition reaction at 120°C. The CO_2_ storage capacity in COF-301-PL was determined by measuring the amount of carbonate produced from the cycloaddition reaction (details in the Supporting Information, Section S9). In the blank experiment, only epichlorohydrin and TBAB were added without any additional adsorbent. As shown in Fig. 4a, under the same experimental conditions for COF-301-PL, in the blank experiment only 3.78 mmol of epichlorohydrin was converted to chloropropene carbonate, achieving a conversion rate of 18.7% (Table S6). The chloropropene carbonate in the blank experiment was primarily produced from residual CO_2_ in the high-pressure reactor and pipelines. After accounting for the blank experiment result, COF-301-PL achieved an additional carbonate yield of 49.9%, corresponding to a CO_2_ adsorption capacity of 6.17 mmol. In contrast, when PEGS was used as the adsorbent, only a low yield of 20.2% was obtained, and PEGS was found to adsorb only 0.25 mmol of CO_2_ (Table S6), which is just 4% of the CO_2_ adsorption capacity of COF-301-PL. This result indicates that PEGS has a weak CO_2_ adsorption capacity and lacks CO_2_ storage capability, consistent with the results confirmed by high-pressure adsorption experiments (Fig. 2a). Based on the above results, the high conversion efficiency of CO_2_ in COF-301-PL can be attributed to its intrinsic porous structure, which temporarily stores adsorbed CO_2_ and gradually releases it under atmospheric pressure, thereby facilitating the conversion of catalytic substrates. Furthermore, although the solid COF-301-Im framework itself exhibited good CO_2_ adsorption capacity, it only achieved a relatively low yield of chloropropene carbonate (20.7%), adsorbing an additional 0.36 mmol of CO_2_. This phenomenon is due to the fact that solid material cannot effectively store gas at atmospheric pressure and thus the rapid desorption of CO_2_ from the solid material during depressurization. In addition, the suspension obtained by directly mixing COF-301 with PEGS showed poor catalytic performance, with a yield of only 28.9%, and an additional CO_2_ adsorption of merely 1.98 mmol. This emphasizes that high-capacity CO_2_ adsorption in PLs cannot be achieved through simple physical mixing, as aggregation and sedimentation of COF particles destroy the porosity of the PL and increase mass transfer resistance. From these experimental results, we conclude that COF-301-PL, with its fluid cavities, demonstrates superior CO_2_ adsorption capacity in multiphase catalytic reactions, significantly outperforming other reported PLs, such as H-ZSM-5 liquid/[P6614][Br] (0.59 mmol@10 bar) [27], 18-C-6-PL (0.43 mmol@10 bar) [50], 15-C-5-PL (0.37 mmol@10 bar) [51] and HCS liquid (0.57 mmol g@10 bar) [32] (Fig. S16). Therefore, COF-301-PL can serve as an outstanding CO_2_ reservoir, significantly enhancing the conversion efficiency of the CO_2_ cycloaddition reaction.

**Figure 5.**
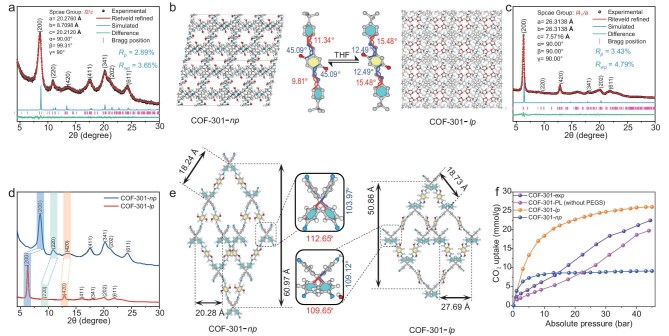
(a) PXRD patterns of the COF-301-*np* phase, along with its Rietveld refinement results and unit cell parameters. (b) The crystal cell structures of COF-301-*np* and COF-301-*lp* phases with the geometric configurations of molecular fragments within the framework. (c) PXRD patterns of the COF-301-*lp* phase, along with its Rietveld refinement results and unit cell parameters. (d) The comparative PXRD patterns of COF-301-*np* and COF-301-*lp* phases. (e) The precise structures of the *dia* topology network in both COF-301-*np* and COF-301-*lp* phases obtained through Rietveld refinement. (f) High-pressure CO_2_ adsorption isotherms of COF-301 and COF-301-PL (without PEGS) compared with simulated CO_2_ adsorption curves of COF-301-*np* and COF-301-*lp* phases.

To confirm whether gas filling induces the dynamically expanding effect of the flexible COF-301-PL and in turn enhances gas adsorption capacity, the simulated and experimental CO_2_ adsorption isotherms and the structures of the porous framework were studied. As shown in the CO_2_ high-pressure adsorption curve of COF-301-PL (Fig. 2a), we found that the incremental adsorption per unit pressure gradually increases with rising CO_2_ filling pressure. Specifically, within the pressure range of 0–10 bar, the slope of the CO_2_ adsorption isotherm for COF-301-PL is 0.116 mmol g⁻¹ bar⁻¹, while in the pressure range of 20–30 bar, the slope increases to 0.221 mmol g⁻¹ bar⁻¹, ∼1.9 times higher than the former. The slope of the CO_2_ adsorption isotherm for PEGS consistently remains in the range of 0.0335 mmol g⁻¹ bar⁻¹ to 0.0369 mmol g⁻¹ bar⁻¹. We infer that the responsive adsorption behavior of COF-301-PL to external pressure changes is due to phase transitions during the expansion of narrow pores in the flexible COF nanocrystals. During CO_2_ filling, the deformation of the COF-301 framework building blocks, displacement between interpenetrated frameworks, and configuration changes of linkages with different bond angles cause a gradual transition of the framework pores from a narrow-pore (***np***) phase to a large-pore (***lp***) phase (Fig. S7). This transition, similar to the swelling of MIL-88 when exposed to water and alcohol compounds, is known as the ‘breathing effect’ [51,52]. Since the CO_2_-driven phase transition occurs under high pressure and the difficult to observe response changes of the liquid material, we used tetrahydrofuran (THF) guest molecules to replace CO_2_ at ambient pressure to demonstrate the existence of the ***np*** to ***lp*** phase transition in the parent COF-301 framework. This was confirmed by PXRD experiments (Fig. 5d). Upon the addition of THF, COF-301 showed significant crystal expansion in its solvated ***lp*** phase, resulting in an increase in unit cell volume by >40% compared to the ***np*** phase. As shown in Fig. 5c, Rietveld refinement provided satisfactory convergence, with the expanded COF-301 space group identified as ***I*4_1_/*a***, having unit cell parameters *a* = *b* = 26.313(8) Å, *c* = 7.5716(0) Å, V = 5242.70 Å^3^ and with factors of *R***_wp_** = 4.79% and *R***_p_** = 3.43% (Tables S3 and S4). Comparing the crystal structures of ***lp*** and ***np*** phases of COF-301, it reveals that the pore window in the ***lp*** phase expands to 8.91 Å, and the unit cell volume increases by 48.83% (***np*** phase unit cell parameters: *a* = 20.276(0) Å, *b* = 8.7098(1) Å and *c* = 20.212(0) Å, V = 3522.44 Å^3^, Fig. 5a, Tables S1 and S2), while the pore volume increases 3.0 times (from 0.183 cm^3^/g to 0.550 cm^3^/g, Table S5 and Fig. S12). These results confirm that COF-301 indeed exists in both ***np*** and ***lp*** phases, and can gradually transition between them during the adsorption/desorption process of guest molecules (the detailed structure is shown in Fig. 5b and e).

Subsequently, the sorption module in the Materials Studio 8.0 software was used to simulate CO_2_ adsorption isotherms on the ***np*** and ***lp*** structures of COF-301 based on the COMPASS force field (Section S7). As shown in Fig. 5f, both the ***np*** and ***lp*** phases of COF-301 reach adsorption saturation before 20 bar, with final CO_2_ saturation adsorption amounts of 9.12 mmol/g and 26.04 mmol/g at 40 bar, respectively. However, the experimental CO_2_ adsorption isotherm of the COF-301 powders (COF-301-exp) shows a gradual increase in CO_2_ adsorption from 0 to 40 bar, with a final CO_2_ adsorption amount of 22.49 mmol/g at 40 bar (Fig. 5f, purple curve). This value is higher than the maximum CO_2_ adsorption of the COF-301-***np*** phase and lower than that of the COF-301-***lp*** phase, indicating that the COF-301 framework undergoes a transition process from the ***np*** phase to the ***lp*** phase during 1–40 bar CO_2_ pressure. Simultaneously, to more intuitively demonstrate the adsorption capacity of the COF host in COF-301-PL under different pressures, the simulated adsorption isotherm of COF-301-PL in Fig. 5f was plotted with the adsorbent mass calculated solely based on the mass of the COF host (marked as COF-301-PL (without PEGS)). As illustrated by the host of the COF-301-PL (without PEGS) curve, the porous host retained adsorption behavior similar to that of COF-301. At a pressure of 43 bar, the CO_2_ adsorption capacity of the porous framework in liquid COF-301-PL also possessed in between the theoretical CO_2_ adsorption capacities of the COF-301-***np*** and COF-301-***lp*** phases (Fig. S11). Therefore, COF-301-PL exhibits the same breathing effect as COF-301 powders, despite its porous host being formed as a liquid with the PEGS. These results provide additional crucial evidence for the CO_2_-driven phase transition process in COF-301-PL.

To validate that the pressure-responsive dynamic phase transition of the flexible porous host in COF-301-PL enhanced CO_2_ adsorption and catalytic efficiency, we saturated COF-301-PL with CO_2_ under various pressures of 1 bar, 5 bar, 10 bar, 15 bar, 20 bar and 25 bar. In the subsequent steps, the adsorbed CO_2_ was utilized for cycloaddition experiments. Except for the saturation CO_2_ pressure, the conditions and procedures of the catalytic experiments were consistent with the previous processes. It should be noted that, under normal circumstances, solid adsorbents typically offer only localized and transient adsorption sites, which make the gas molecules on the solid surface more prone to rapid desorption when external pressure decreases [53–56]. However, according to Henry's law, the fluid medium resistance in PLs slows down the diffusion of dissolved gases [57], preventing gas molecules from immediately and fully desorbing, and instead causing them to be released gradually and slowly from the liquid. Additionally, during the process of gas escaping from the liquid, the surface tension and internal convection effects of the liquid provide an additional energy barrier, further delaying the desorption process [58,59]. Therefore, even after reaching adsorption saturation and returning rapidly to atmospheric pressure, the CO_2_ dissolved in COF-301-PL typically does not undergo significant desorption. The catalytic experimental results of COF-301-PL with CO_2_ at different equilibration times further confirm the slow-release process of CO_2_ in COF-301-PL (Fig. S15, Tables S8 and S9). As shown in Fig. 4b, the catalytic CO_2_ conversion rate for PEGS remained nearly constant (0.25 ± 0.05 mmol) at different pressures. This is primarily attributed to its inherently low CO_2_ adsorption capacity and the absence of intrinsic pores capable of dynamic expansion. Therefore, the catalytic experiment results further confirm that the CO_2_ adsorption amount in the PEGS system remains almost unchanged within the pressure range of 1 to 25 bar, as illustrated by the CO_2_ adsorption curve of PEGS (Fig. 3a). In contrast, under the same conditions, the conversion rate of reactants in COF-301-PL significantly increases with the rise in CO_2_ pressure. As shown in Fig. 4c, the additional CO_2_ adsorption capacity of COF-301-PL increased from 0.28 mmol at 1 bar to 8.97 mmol at 25 bar of CO_2_ pressure. As shown in Fig. 4d, by comparing the experimental results for PEGS and COF-301-PL at 1 bar and 25 bar CO_2_ pressures, respectively, we observed that the CO_2_ adsorption per unit pressure in PEGS decreased significantly as the CO_2_ pressure increased (0.11 mmol/bar at 1 bar and 0.0112 mmol/bar at 25 bar). In contrast, the CO_2_ adsorption per unit pressure in COF-301-PL continued to increase with the rising pressure (0.284 mmol/bar at 1 bar and 0.359 mmol/bar at 25 bar, Table S7). Based on the above results, we infer that the additional increase in CO_2_ adsorption capacity within COF-301-PL originates from the dynamic expansion in the cavities of the porous hosts with increasing pressure, rather than from the adsorption of CO_2_ by the PEGS guest molecules. This phenomenon of pore expansion induced by rising pressure is reported for the first time in PL materials. From a technical perspective, COF-301-PL not only enhances mass transfer efficiency in heterogeneous catalytic reactions by reducing phase transfer processes but also enables the dynamic response of the pore cavities in PLs to external gas pressure environments. This has significant implications for heterogeneous catalysis in high-pressure gas environments.

## CONCLUSION

In conclusion, we successfully synthesized COF-301-PL, a COF liquid characterized by stability and permanent porosity. By employing surface engineering strategies, a stable corona-canopy electric double layer was constructed between COF nanoparticles and PEGS guest molecules, resulting in COF-301-PL with stable and fluid cavities. Molecular dynamics and density functional theory calculations jointly confirmed the formation of the corona-canopy layer and demonstrated that the electrostatic interactions within this structure effectively contribute to COF-301-PL's resistance to solvent intrusion, thereby maintaining its long-term stability and porosity. Furthermore, due to the intrinsic flexibility of the porous framework, we discovered for the first time that under high-pressure conditions, COF-301-PL exhibits a dynamic pore expansion from the narrow-pore (***np***) to large-pore (***lp***) phase as CO_2_ molecules are incorporated, thereby providing additional pore volume to enhance adsorption capacity. The comparison of high-pressure CO_2_ adsorption isotherms between COF-301 powder and COF-301-PL, along with adsorption simulations, corroborated the occurrence of this dynamic expansion in COF-301-PL, marking the first report of such a phenomenon in PLs. Consequently, COF-301-PL can serve as an efficient CO_2_ micro-storage device, which enhances mass transfer efficiency by minimizing phase transfer processes, thus facilitating the cycloaddition of CO_2_ with liquid epoxide substrates to efficiently produce cyclic carbonates. This study not only advances the field of PLs but also highlights the transformative potential of COF liquids in a wide range of industrial applications, from energy to catalysis. The development of durable and stable COF liquids holds promise for innovations in fluid processing and catalytic technologies.

## Supplementary Material

nwaf032_Supplementary_Data
