# Supplementary material for: Porous covalent organic framework liquid for boosting CO2 adsorption and catalysis via dynamically expanding effect
Source: Natl Sci Rev. 2025 Jan 24;12(3):nwaf032. doi: 10.1093/nsr/nwaf032 (PMC11963760; doi:10.1093/nsr/nwaf032)
Supplement: nwaf032_Supplementary_Data [file nwaf032_supplementary_data.pdf]

Supporting Information

Porous Covalent Organic Framework Liquid for Boosting CO_2 Adsorption and Catalysis via Dynamically Expanding Effect

Zi-Ao Chen,[1,3] Lei Zou,[1,2] Rong Cao[1,2,3] and Yuan-Biao Huang*[1,2,3]

[1]State Key Laboratory of Structural Chemistry Fujian Institute of Research on the Structure of Matter, Chinese Academy of Sciences Fuzhou, 350002 (China)

[2]Fujian Science & Technology Innovation Laboratory for Optoelectronic Information of China Fuzhou, Fujian, 350108 (P. R. China)

[3]University of Chinese Academy of Science Beijing 100049 (China)

*Corresponding authors. E−mails: ybhuang@fjirsm.ac.cn

Yuan-Biao Huang orcid.org/0000-0003-4680-2976

Contents

Section 1. Materials and Instruments 3

Section 2. Synthesis of monomers, COF-301, COF-301-Im and COF-301-PL. 5

Section 3. Electron microscope image of COF-301-PL 12

Section 4. Structure simulations and Ritveld refinements of COF-301 13

Section 5. Molecular Structure Simulations and Visualizations 19

Section 6. Molecular dynamics (MD) and CO_2 adsorption simulation 21

Section 7. Porosity and Specific Surface Area Analysis. 26

Section 8. Structure and performance characterization 28

Section 9. Details of catalytic cycloaddition of epoxide and carbon dioxide ... 30

Section 10. Reference 36

Section 1. Materials and Instruments

Chemicals

Acetic acid, acetone, tetrahydrofuran, ethanol, ethyl acetate, and petroleum ether from Sinopharm National Medicine Corporation. 1,2-Dichlorobenzene (*o*-DCB), N,N-dimethylacetamide (DMAc) from J&K Scientific Technology Co., Ltd. 4-(4-aminophenyl) methane (TAM), 2,5-dihydroxy-1,4-benzaldehide (DBA), NaH, KBr, Poly(Ethylene Glycol Methyl Ether) (M.W.=550), imidazole, (3-iodopropyl)trimethoxysilane, *p*-toluenesulfonyl chloride (TsCl), and tetrabutylammonium bromide (TBAB) are all from Adamas (China). Poly(ethylene glycol)-tailed sulfonate (PEGS) is from Sigma-Aldrich Co., Led. Except for Poly(Ethylene Glycol Metal Ether) (M.W.=550),other chemicals were commercially available and used without further purification.

Instruments

Powder X-ray diffraction patterns (PXRD) were recorded on a Rigaku Dmax 2500 diffractometer equipped with Cu-Kα radiation (λ= 1.54056 Å) over the 2θ range of 3- 30° with a scan speed of 3° min^{-1} at room temperature. The ^{1}H NMR was performed at AVANCE III Bruker Biospin spectrometer, operating at 400 MHz. Solid-state ^{13}C CP/MAS NMR was performed on a Bruker SB Avance III 500 MHz spectrometer with a 4-mm double-resonance MAS probe. Fourier transform infrared (FT-IR) spectra were recorded with KBr pellets using Perkin-Elmer Instrument. The thermogravimetric analysis (TGA) was performed on a TA SDT Q600 instrument, and the as-synthesized crystalline samples were heated at a rate of 5 °C/min up to 1000 °C and then cooled to room temperature under N_2 atmosphere. The differential scanning calorimetry (DSC) traces were recorded by a DTA404PC instrument. The rheological properties of COF-301-PL were measured on TA Discovery Hybrid Rheometer-2 at 298 K. Nitrogen adsorption and desorption isotherms were measured at 77 K using a Micromeritics ASAP 2460 system. The samples were degassed at 393 K for 10 h before the measurements. Specific surface areas were calculated from the adsorption data using Brunauer-Emmett-Teller (BET) equation. The calculation of the pore size distribution was

done using the quenched-solid density functional theory (QS-DFT) equilibrium model. CO_2 high-pressure absorption measurements were acquired using a BSD-PH High Pressure Gas Sorption Analyzer. The gas chromatography (GC) measurements were performed on a G7890A-GC equipped with a HP-5 column, FID detector and autosampler. The morphologies of COF-301, COF-301-Im and COF-301-PL were studied using a FEIT 20 transmission electron microscope (TEM) were performed on a JEOL JSM-7500F operated at an accelerating voltage of 3.0 kV. X-ray photoelectron spectroscopy (XPS) measurements were performed on Thermo ESCALAB 250 spectrometer using non-monochromatic Al Kα X-rays as the excitation source and choosing Carbon 1s (284.8 eV) as the reference line.

Section 2. Synthesis of monomers, COF-301, COF-301-Im and COF-301-PL.

Section S2.1 Synthesis of PEG-Im-Si(OCH$_3$)$_3$ molecular.

PEG-Im-Si(OCH$_3$)$_3$ was synthesized stepwise, as illustrated in the scheme below.

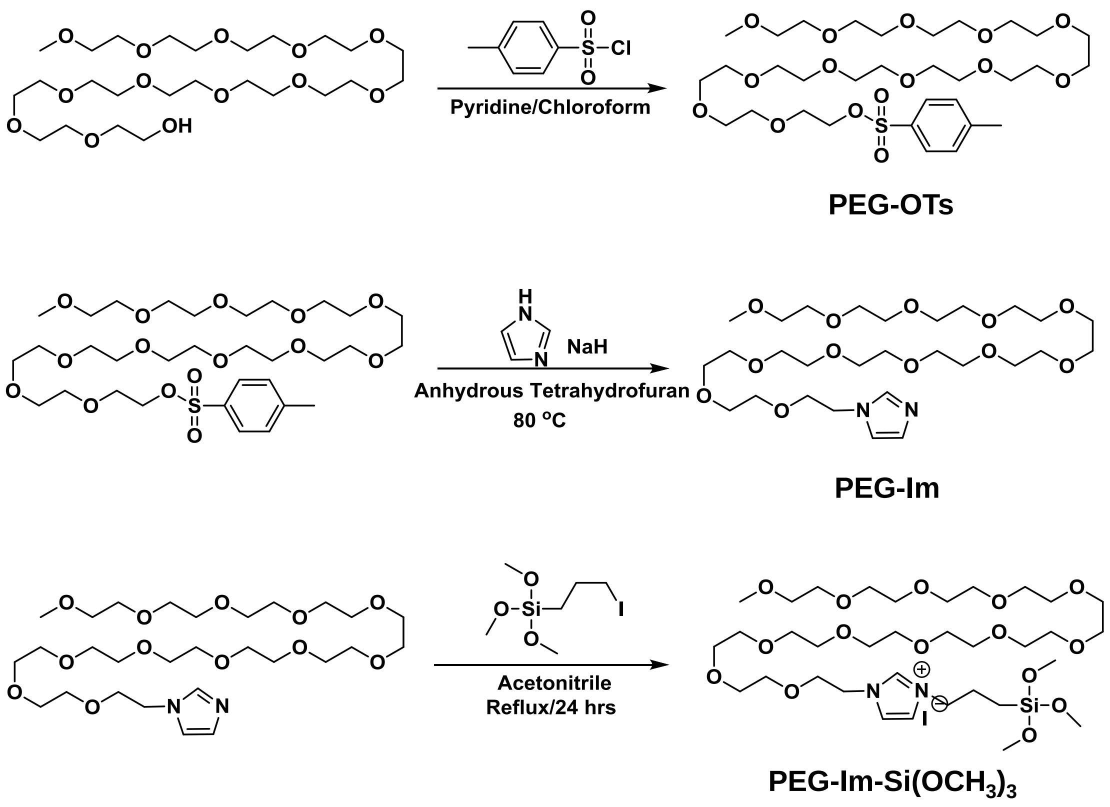

Synthesis of PEG-OTs molecular.

1. PEG-OTs, synthesized through the sulfonation reaction of purified Poly(ethylene glycol methyl ether) (M.W.=550, denoted as PEG-550), involves a series of steps. Initially, 40 mL of commercially acquired PEG-550 is purified through column chromatography with a petroleum ether/dichloromethane=10:1. The middle-stage eluate is collected, and the removal of petroleum ether and dichloromethane is achieved through rotary evaporation, yielding a colorless and transparent liquid product.

2. Weigh 11g (20 mmol) of purified PEG-550 and dissolve it in 40 mL of pyridine.

3. Dissolve 7.6g (40 mmol) of p-toluenesulfonyl chloride in 60 mL of chloroform, then add it dropwise to the pyridine solution at 0°C. After the addition is completed, raise the reaction temperature to 30 °C and stir for 24 hours.

4. Add 40 mL of deionized water to step 3 at room temperature and stir for 24 hours.

5. Add 25 mL of concentrated hydrochloric acid to step 4 at room temperature and stir for 24 hours.

6. Extract the solution in step 5 with dichloromethane (150 mL x 3) and wash the organic phase with a saturated NaCl aqueous solution.

7. Dry the organic phase overnight with anhydrous Na_2SO_4, and then evaporate all solvents using a rotary evaporator to yield a light-yellow viscous liquid.

8. Column chromatography was conducted using dichloromethane/ethyl acetate (4:1) as the eluent, resulting in a viscous light-yellow liquid, PEG-OTs (7.43 g, yield=52%).

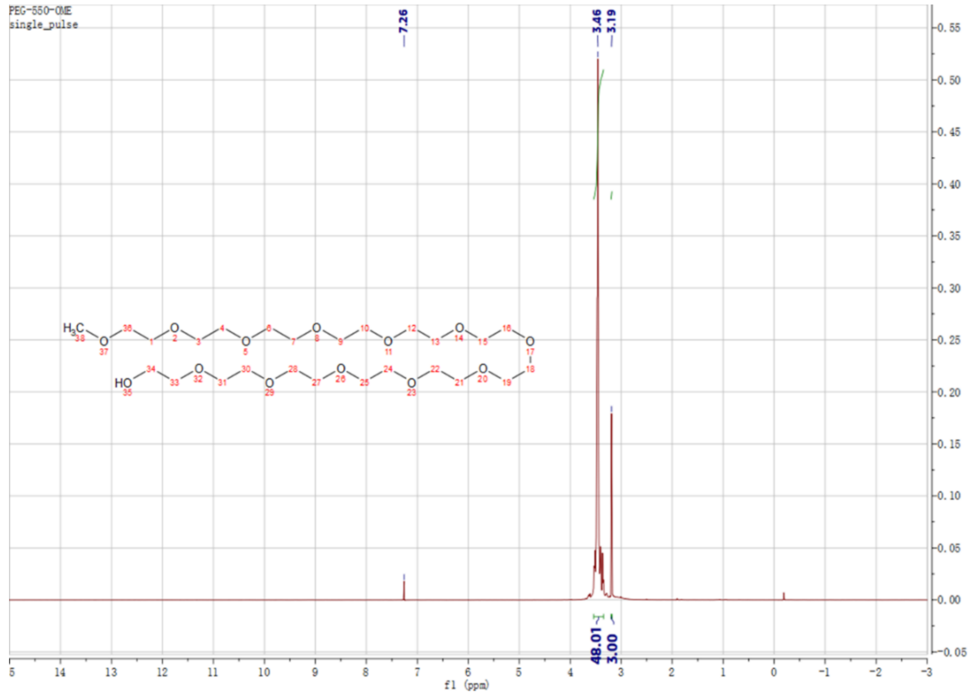

Figure S1. ^{1}H NMR spectrum of **PEG-550** (400 MHz, Chloroform-*d*) δ 3.46 (m, 48H), 3.19 (s, 3H).

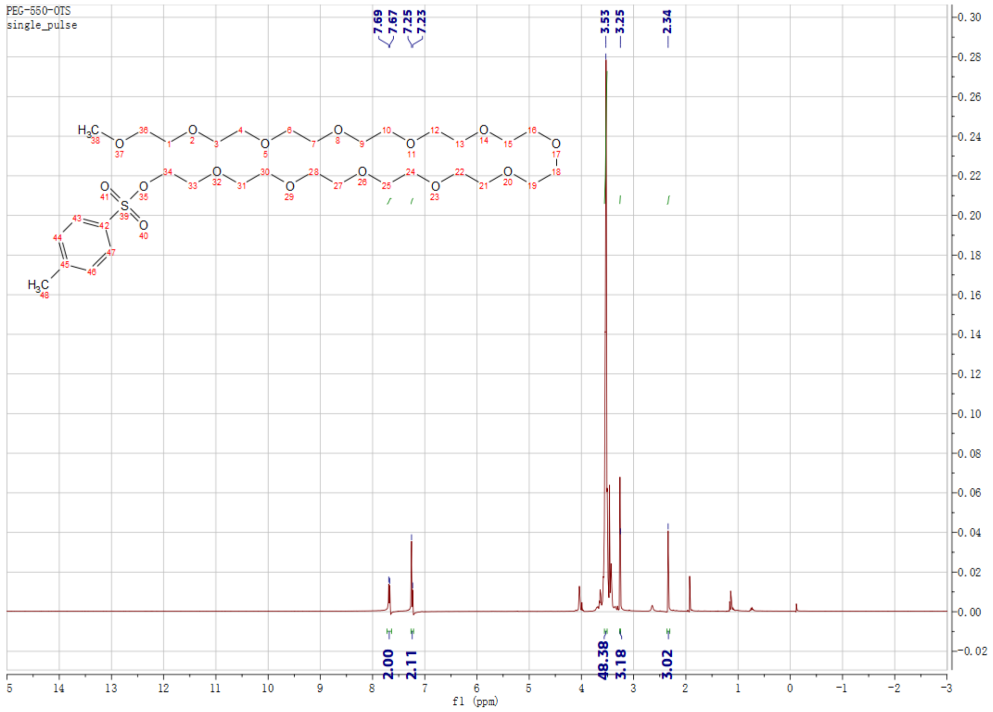

Figure S2. *J* = 8.6 Hz **S2.** ^{1}H NMR spectrum of **PEG-OTs** (400 MHz, Chloroform-*d*) δ 7.68 (d, *J* = 8.3 Hz, 2H), 7.24 (d, , 2H), 3.53 (m, 48H), 3.25 (s, 3H), 2.34 (s, 3H).

Synthesis of PEG-Im molecular.

1. After dispersing 456 mg of sodium hydride (11.4 mmol, 60%) in 40 mL anhydrous THF, the temperature of the reaction system was lowered to -20°C.

2. Dissolve 704 mg of imidazole (10.4 mmol) in 30 mL anhydrous tetrahydrofuran, then slowly add it dropwise to the mixture in 1 over 20 minutes, stirring for an additional 30 minutes after the completion of the addition.

3. Maintain the reaction temperature at -20 degrees Celsius, slowly add 7.1 g (10.0 mmol) of PEG-OTs, and after the addition, raise the reaction temperature to 80 degrees Celsius for 4 hours.

4. After the reaction, add the solution in 3 to 40 mL of deionized water and extract the organic phase with dichloromethane.

5. Collect the organic phase, wash it twice with saturated NaCl aqueous solution, and finally dry the organic phase over anhydrous Na_2SO_4 for 24 hours.

6. Purify the product by column chromatography using elution with ethyl acetate/dichloromethane = (1:1), resulting in a viscous light-yellow liquid, PEG-Im (5.5g, yield = 90.1%).

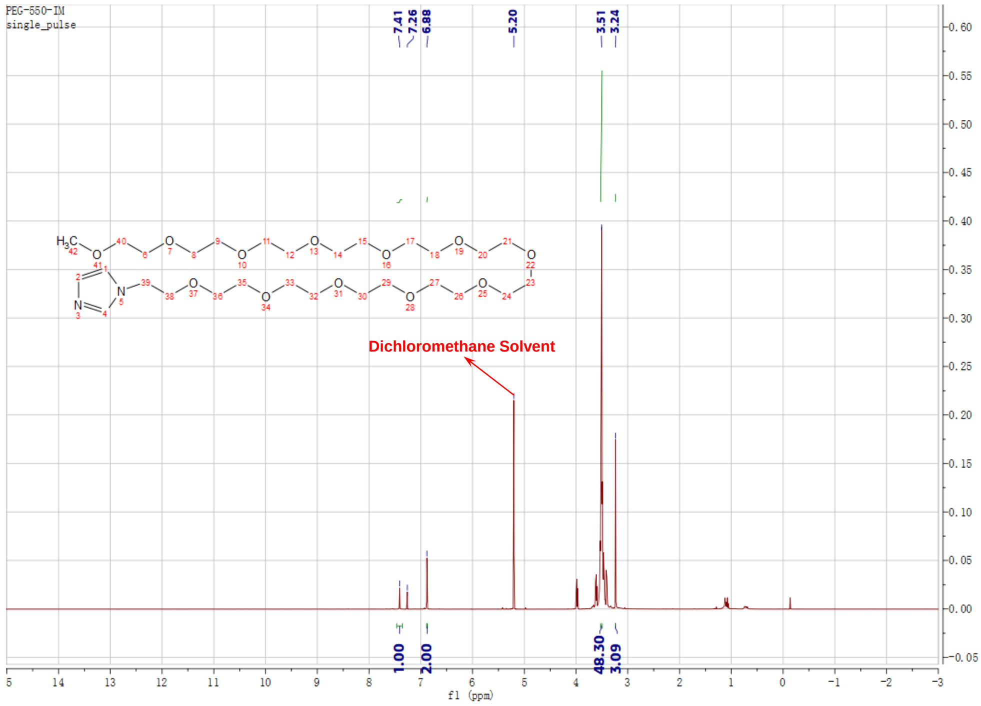

Figure S3. ^{1}H NMR spectrum of **PEG-Im** NMR (600 MHz, Chloroform-d) δ 7.19 (s, 1H), 6.68 (s, 1H), 6.62 (s, 1H), 3.27 (m, 48H), 2.99 (s, 3H).

Synthesis of PEG-Im-Si(OCH$_3$)$_3$ molecular.

1. Dissolve 5.5 g of PEG-Im (9 mmol) and 2.89 g of (3-iodopropyl)trimethoxysilane in 40 mL of acetonitrile.

2. After refluxing for 24 hours, the solvent was removed by rotary evaporation to obtain the crude product, a viscous brownish-yellow liquid.

3. The crude product was washed repeatedly with icy ether until the ether became completely colorless, resulting in a viscous dark brown liquid, PEG-Im-Si(OCH$_3$)$_3$ (7.68 g, yield = 95%).

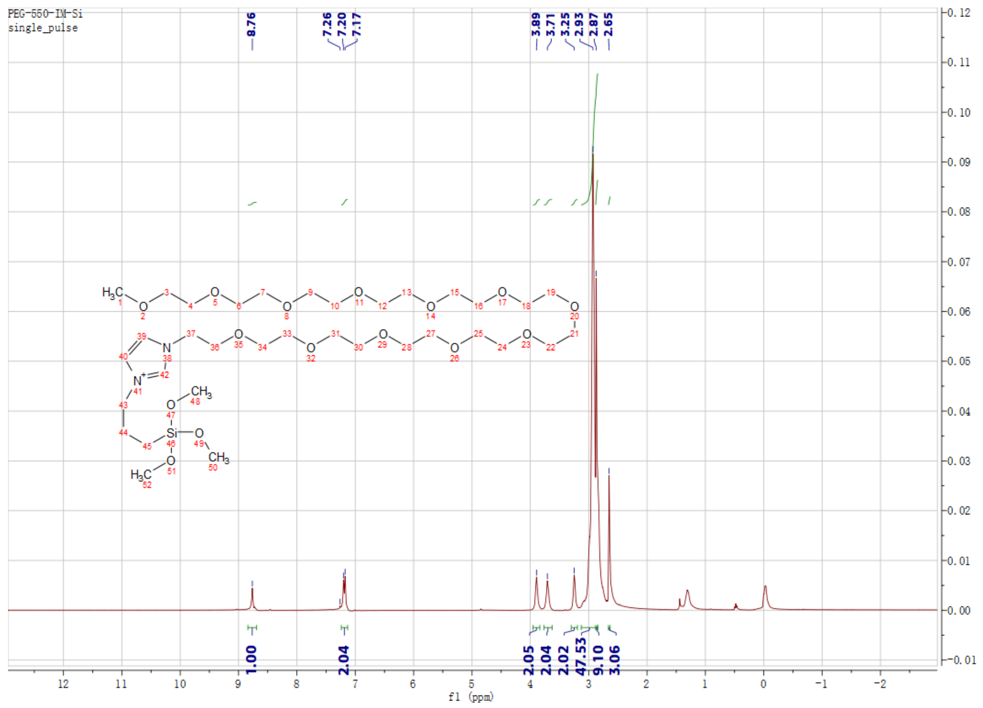

Figure S4. ^{1}H NMR spectrum of **PEG-Im-Si(OCH_3)$_3$** NMR (400 MHz, Chloroform-d) δ 8.76 (s, 1H), 7.19 (d, J = 9.9 Hz, 2H) , 3.89 (s, 3H), 3.71 (s, 3H), 3.25 (s, 3H), 2.93 (m, 48H), 2.87 (s, 9H), 2.65 (s, 3H).

Section S2.2 Synthesis of COF-301, COF-301-Im and COF-301-PL.

Synthesis of COF-301.

15.2 mg **TAM** (0.04 mmol) and 13.2 mg **DBH** (0.08 mmol) were added in a mixture of *o*-DCB / DMAc / 6 M acetic acid (0.30 mL / 0.70 mL / 0.10 mL) in a Pyrex tube. The tube with the mixture was placed and sonicated for at least 10 min, and then degassed through three freeze–pump–thaw cycles, which was tightly capped and then heated at 120 ºC for 3 days before the next isolation procedure. Orange solid powder was isolated by centrifugation and subsequently be washed by Soxhlet extractor with anhydrous *n*-Hexane, anhydrous tetrahydrofuran and anhydrous acetone, respectively. Finally, the as-synthesized COFs were obtained after drying under vacuum at 80 °C for 10 h to give 22.9 mg (91% isolated yield) orange powder, which is insoluble in common organic solvents.

To further reduce the particle size of COF-301, the Soxhlet-extracted COF-301 powder was dispersed in THF, followed by the addition of 5% (wt.) aniline. The mixture was ultrasonically dispersed for 40 minutes. The product was then separated by centrifugation and washed three times with THF to completely remove the aniline.

Element Analysis of activated COF-301: Calculated: C, 77.35%; H, 3.80%; N, 8.80% O, 10.05%. Found: C, 77.02%; H, 4.47%; N, 8.13%.

Synthesis of COF-301-Im.

320 mg of COF-301 nanoparticle (with a phenolic hydroxyl content of 4 mmol) was dispersed in 200 ml of anhydrous ethanol and sonicated for 30 minutes. Under vigorous stirring, 5.4g of PEG-Im-Si(OCH_3)$_3$ (6 mmol) was added, and the mixture was reacted at 40 ^{o}C for 24 hours. After the reaction, COF-301-Im crude product was obtained by centrifugation. Subsequently, the crude COF-301-Im was Soxhlet extracted with acetone, tetrahydrofuran, and anhydrous ethanol for 24 hours each to remove excess PEG-Im-Si(OCH_3)$_3$ molecules. Finally, the solid obtained after Soxhlet extraction was vacuum dried at 80 ^{o}C for 12 hours to remove all residual solvents, resulting in a brownish paste-like solid (1.06g).

Synthesis of COF-301-PL.

COF-301-Im (1.0 g) was dispersed in ethanol (50 mL) and sonicated for 30 minutes. Then, under vigorous stirring, a PEGS solution in ethanol (25 mL, 45%) was added to the suspension of COF-301-Im. Figure S8. Center-of-mass SDF of anions (blue) and cations (red) around the COF-301 (a-c) and COF-301-Im (d-f) was heated and refluxed for 12 hours, after which ethanol was removed via rotary evaporation. The remaining liquid was then transferred into a dialysis bag with a molecular weight cutoff of 550 and washed 2-3 times with warm acetone. During this process, excess PEGS was extracted, and K^+ and I^- were removed to complete the ion exchange. Finally, the ion-exchanged solution was thoroughly

evaporated at 100° C to remove acetone and subsequently dried overnight in a vacuum environment at 80° C, yielding a brownish-yellow liquid, COF-301-PL.

Section 3. Electron microscope image of COF-301-PL

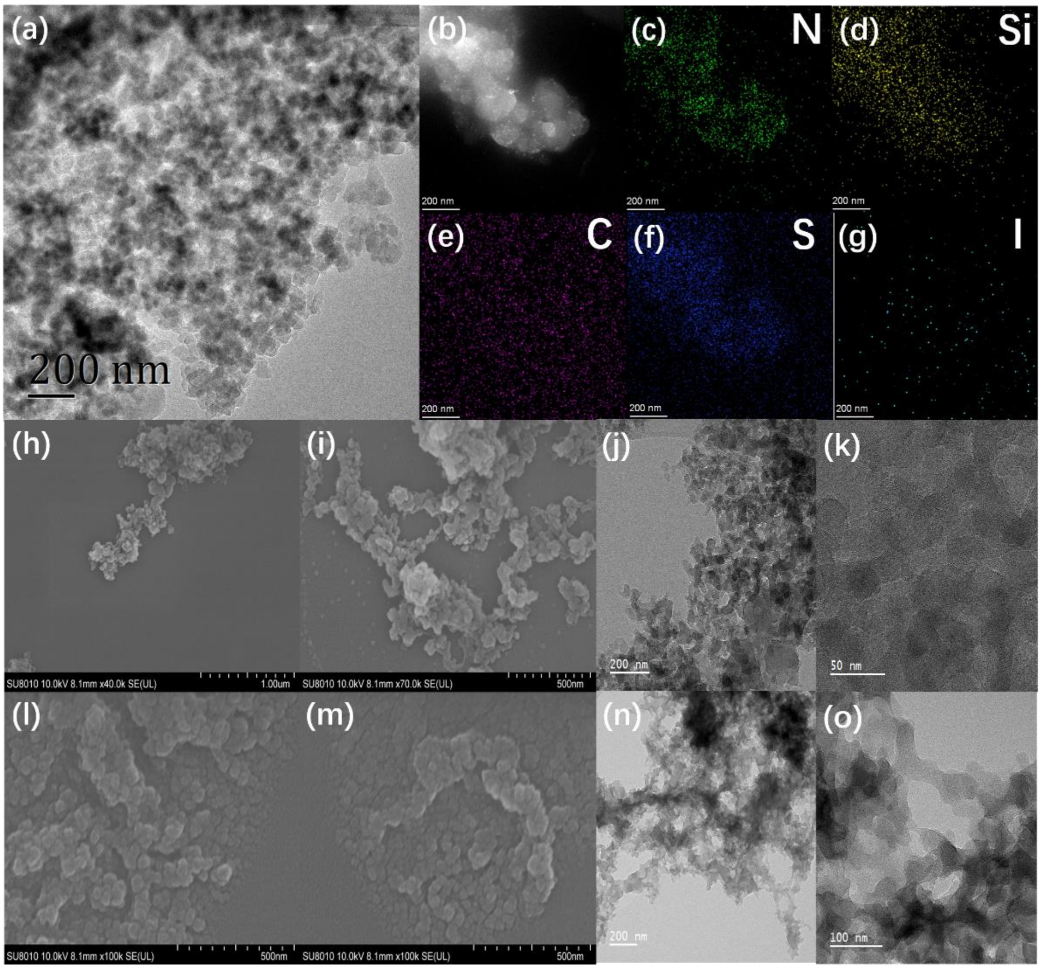

Figure S5. (a) HR-TEM images of COF-301-PL. High-angle annular dark field-STEM image of COF-301-PL (b), with element mapping for N (c), Si (d), C (e), S (f) and I (g). The weight percentages and atomic percentages of the elements in COF-301-PL are as follows: C (78.29% / 68.56%), N (4.88% / 4.98%), O (12.01% / 14.00%), S (2.10% / 4.91%), K (2.47% / 7.04%), and Si (0.24% / 0.50%). The SEM images of COF-301 (h and i) and COF-301-Im (l and m). The HR-TEM images of COF-301 (j and k) and COF-301-Im (n and o).

Section 4. Structure simulations and Ritveld refinements of COF-301

PXRD data collection

PXRD data collection PXRD measurements were carried out using a Rigaku Miniflex 600 diffractometer in graphite monochromator filtered Cu Kα focused radiation (1.5416 Å) at 600 W (40 kV, 15 mA) power, which was equipped with a D/teX Ultra2 detector. The best counting statistics were achieved by collecting samples using a 0.01° 2θ step scan from 3–30° with exposure time of 5 s per step. The measurement was performed at room temperature and atmospheric pressure.

Structural model

Before the simulation, the structure was firstly optimized in Gaussian 16 package by calculations at B3LYP/6-31G d(p) level. The structural model of all COFs were executed by using the Materials Visualizer module of Materials Studio software (Material Studio ver. 8.0, Accelrys Software Inc.). Upon completion of the structural model, an energetic minimization was performed using the universal force field implemented in the Forcite module of Materials Studio 8.0 Software. During this process, the unit cell parameters were optimized until proper convergence was achieved (energy convergence criteria were set at 10^{-4} kcal mol^{-1}).

PXRD patterns refinement

For all experimental powder patterns, Rietveld refinement was performed for the full-profile pattern fitting from 2θ = 3-30° . The Reflex module in Materials Studio 8.0 software was used to incrementally optimize parameters, including peak profile, zero shift, background, cell parameters, Berar-Baldinozzi asymmetry correction parameters, preferred orientation, and global isotropic temperature factors. This approach ensured that the calculated PXRD patterns matched the experimental PXRD patterns satisfactorily. As demonstrated by the fitting, the refinement converged with reasonable residual values, yielding the final cell parameters.

Table S1. Rietveld refined cell parameters for COF-301-*np* phase.

Parameters	COF-301-***np***
Moiety Formula	$C_{138}H_{99}N_{16}O_{16}$
Formula Weight	2236.55
Crystal system	Monoclinic
Space group	I_2/C (15)
Symmetry	Body-Centered Tetragonal
Z	4
Density(g/cm^3)	1.2082
a (Å)	20.2760
b (Å)	8.7098
c (Å)	20.2120
α°	90.0000
β°	99.3080
γ°	90.0000
Unit Cell Volume (Å^3)	3522.44
Refined R_p factors (%)	2.89
Refined R_{wp} factors (%)	3.65

Table S2. Atomic coordinates and initial unit cell parameters of COF-301-*np* phase.

COF-301-***np*** phase			
***I*₂/C** (15) group			
**Atom**	**x/a**	**y/b**	**z/c**
C8	0.6808	-0.4643	0.7199
N1	0.6944	-0.3464	0.7579
O1	0.7007	-0.6764	0.6185
C5	0.6512	-0.2143	0.7503
C6	0.5965	-0.2105	0.6995
C7	0.5504	-0.093	0.6971
C2	0.55818	0.0209	0.7455
C3	0.6125	0.018	0.7966
C4	0.6589	-0.0996	0.7988
C9	0.7165	-0.611	0.7353
C10	0.7251	-0.7123	0.68371
C11	0.7586	-0.8508	0.6984
N2	0.5367	0.6424	0.9208
C15	0.53	0.4981	0.8867
C16	0.5835	0.4393	0.8596
C17	0.5756	0.3082	0.8207
C12	0.5145	0.235	0.80934
C13	0.4608	0.2957	0.8348
C14	0.469	0.4243	0.8754
C18	0.4923	0.6901	0.954
C19	0.4978	0.8457	0.9825
O2	0.6128	0.8578	0.9696
C20	0.5565	0.9279	0.9847
C21	0.4415	0.918	0.998
H1c3	0.61808	0.09691	0.8302
H1c4	0.69669	-0.10189	0.83422
H1c6	0.59066	-0.28949	0.66591
H1c7	0.51274	-0.09046	0.6617

Atom	x/a	y/b	z/c
H1c8	0.64694	-0.45695	0.68094
H1c13	0.41766	0.24843	0.82423
H1c14	0.43252	0.46191	0.89571
H1c16	0.62602	0.48997	0.86802
H1c17	0.61246	0.26809	0.80174
H1c18	0.45563	0.62495	0.96035
H1c11	0.76456	-0.91997	0.66285
H1c21	0.40081	0.86108	0.99669
H1o1	0.68472	-0.56497	0.61119
H1o2	0.60265	0.75099	0.94577
C1	0.5	0.1284	0.75

Table S3. Rietveld refined cell parameters for COF-301-*lp* phase.

Parameters	COF-301-***lp***
Moiety Formula	$C_{138}H_{99}N_{16}O_{16}$
Formula Weight	2236.55
Crystal system	Tetragonal
Space group	$\boldsymbol{I4_1/a}$ (16)
Symmetry	Body-Centered Tetragonal
Z	4
Density(g/cm^3)	0.7090
a (Å)	26.3138
b (Å)	26.3138
c (Å)	7.5716
α°	90.0000
β°	90.0000
γ°	90.0000
Unit Cell Volume (Å^3)	5242.70
Refined R_p factors (%)	3.43
Refined R_{wp} factors (%)	4.79

Table S4. Atomic coordinates and initial unit cell parameters of COF-301-*lp* phase.

COF-301-***lp*** phase			
I 4_1/***a*** (16) group			
**Atom**	**x/a**	**y/b**	**z/c**
C002	0.50041	0.79653	0.24786
C003	0.46238	0.83445	0.24597
H003	0.43109	0.83413	0.15388
N004	0.49319	0.91034	0.64017
C005	0.46269	0.87315	0.37072
H005	0.43304	0.90172	0.36945
C006	0.49931	0.874	0.50304
C007	0.53776	0.79863	0.38107
H007	0.56663	0.76938	0.38941
C008	0.53732	0.83679	0.50699
H008	0.56573	0.83567	0.60913
C009	0.51128	0.95804	0.89519
C00A	0.52386	0.91827	0.77039
H00A	0.56065	0.90017	0.77957
C1	0.45119	1.00582	1.06947
H1	0.41364	1.00901	1.12686
C1AA	0.46188	0.9636	0.96718
O	1.32232	1.17311	2.69847
H	1.35323	1.1827	2.6271
C001	0.5	0.75	0.125

Section 5. Molecular Structure Simulations and Visualizations

Structure Calculations and Simulations

The simulation of the structure for COF-301-***np*** and COF-301-***lp*** was modeled and optimized with Forcite package on Geometry Optimization task based on Materials Studio 8.0 software. The B3LYP hybrid functional were adopted for all molecular (CO_2, PEGS and PEG-Im-$Si(OCH_3)_3$) structural optimization calculations by means of the Gaussian16 program, and the 6-31G (d, p) set as the basis sets. The molecular volume and electrostatic potential of monomers were analyzed by the Multiwfn package, and visualized by VMD program.

Non-Covalent Interaction （NCI）visualized

The non-covalent interaction (NCI) analysis of intermediates was calculated by B3LYP/def2-TZVP basis set with DFT-D3 correction through ORCA 5.0.4 software.[1-2] The display of NCI was performed with the Multiwfn software and visualized by VMD package.[3-4]

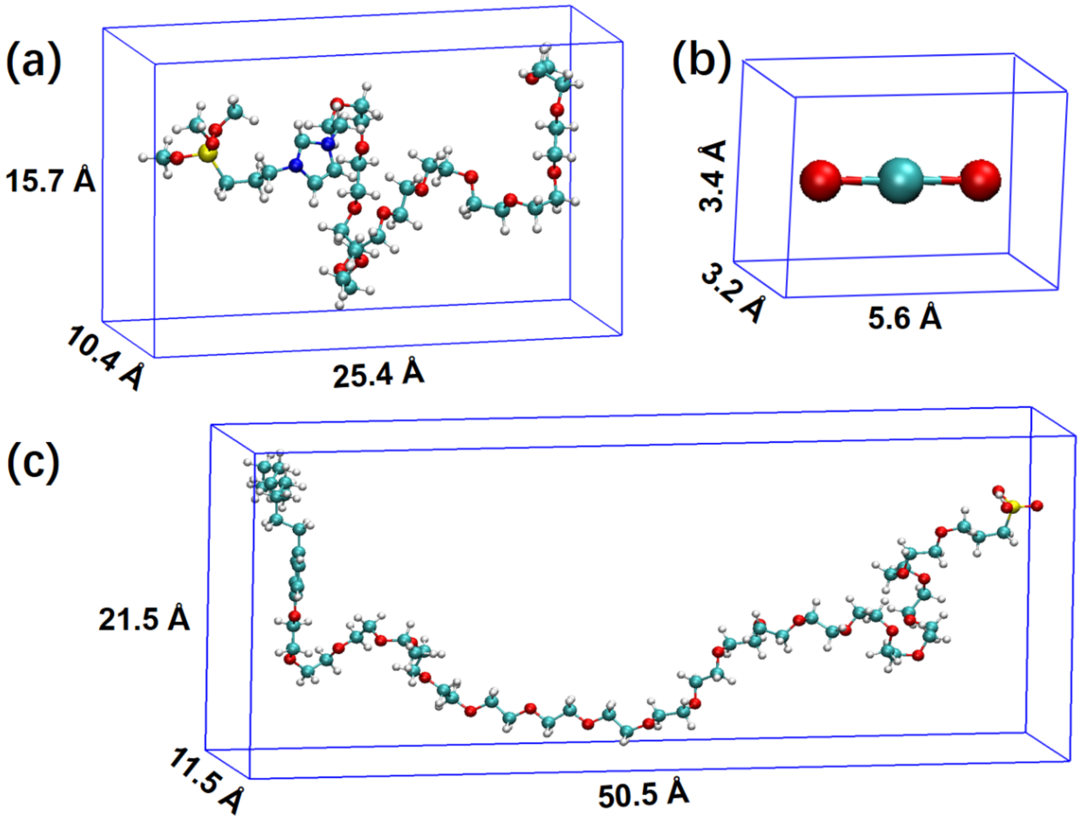

Figure S6. The sizes of PEG-Im-Si(OCH_3)$_3$ (a), PEGS (b) and CO_2 (c) calculated by density functional theory (DFT).

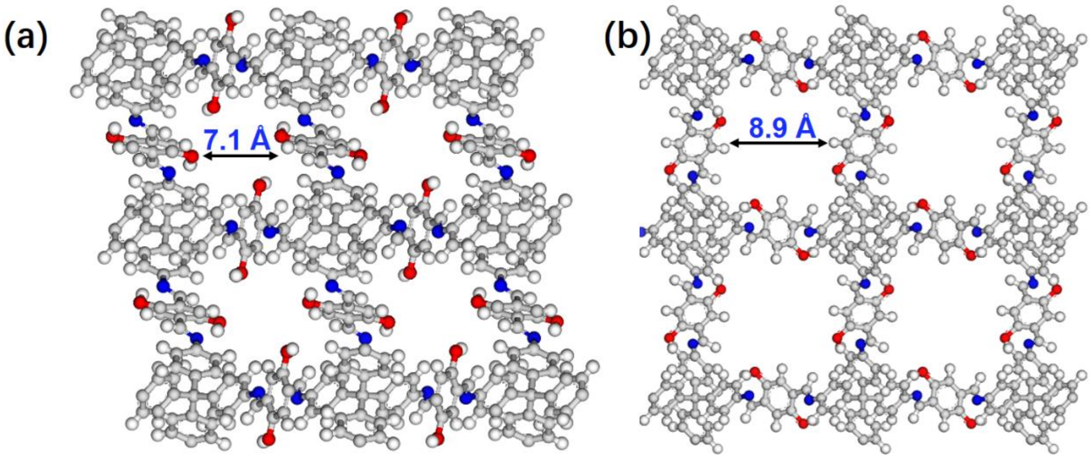

Figure S7. The pore window sizes of COF-301-***np*** (a) and COF-301-***lp*** (b).

Section 6. Molecular dynamics (MD) and CO_2 adsorption simulation

Molecular dynamics (MD) simulations.

The atomistic MD simulations were performed with the GROMACS 2024.2 software package.[5] Cubic boxes containing 1 COF model compound and 200 ion pairs were constructed. Periodic boundary conditions were applied with long-range interactions handled with Particle-Mesh Ewald summations.[6] The energies of systems were minimized using the steepest descent algorithm until the steepest descents converged to $F_{max} < 10$. Equations of motion were integrated using the leapfrog algorithm with a time step of 2 fs. The system was built and imitated using the following procedure:

(1) 5 ns of simulation at 800 K and 100 bar to make molecules close together quickly;

(2) 10 ns of simulation at 800 K and 1 bar, then cooling down to 300 K in 5 ns;

(3) 20 ns of equilibration at 300 K and 1 bar (equilibration).

The Berendsen method was applied for the control of pressure, and the velocity rescaling method with a stochastic term (v-rescale) was applied for the control of temperature. All covalent hydrogen bonds were constrained using the LINCS algorithm and a cutoff range for the short-range electrostatics was set to 13 Å. The general AMBER force field (GAFF) was used for all compounds based on optimized structures.[7]

The geometry of COF-301-Im model compound was optimized at B3LYP/6-31 G(d,p) level by Gaussian 16 software. The geometry of COF was optimized by Forcite forcefield, and during the MD process, the structural position is restricted in the center of cell. Restrained electrostatic potential (RESP) atomic charges for COF-301-Im or MMFF94 atomic charges for COF were calculated by Multiwfn software and OpenBabel software based on the optimized structures.[4, 8-9]

Radial distribution functions (RDF) and spatial distribution functions (SDF) were computed using the TRAVIS program.[10-11]

The cubic box is outlined in blue. In the MD snapshots, COF-301 and COF-301-Im fragments are depicted by the Corey-Pauling-Koltun (CPK) model. The colors of different atoms in COFs fragments are as follows: hydrogen (white), oxygen (red), nitrogen (blue), carbon (gray), and silica (purplish red).

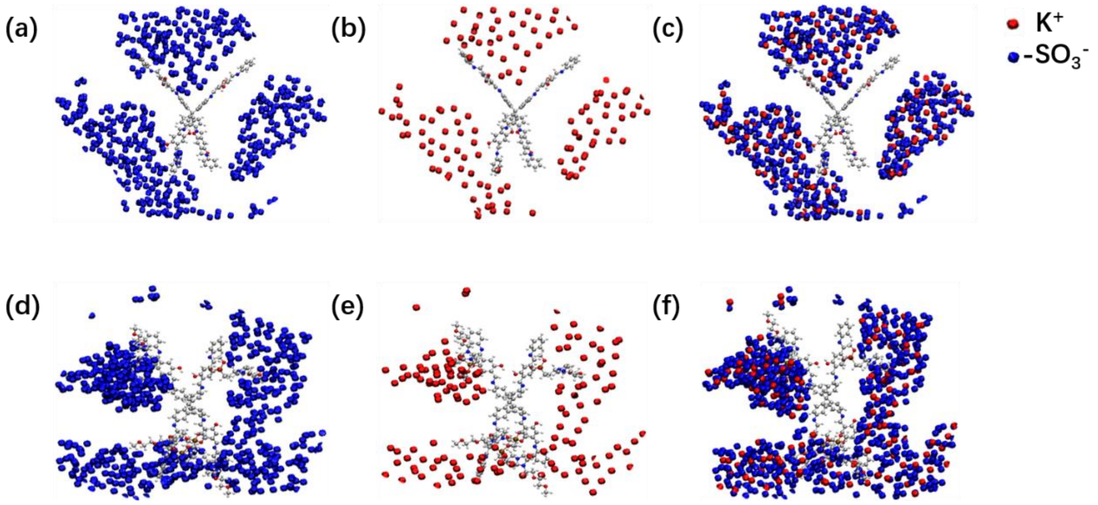

Figure S8. Center-of-mass SDF of anions (blue) and cations (red) around the COF-301 (a-c) and COF-301-Im (d-f). Blue and red represent the isosurfaces of anions ($PEGSO_3^-$) and cations (K^+), respectively.

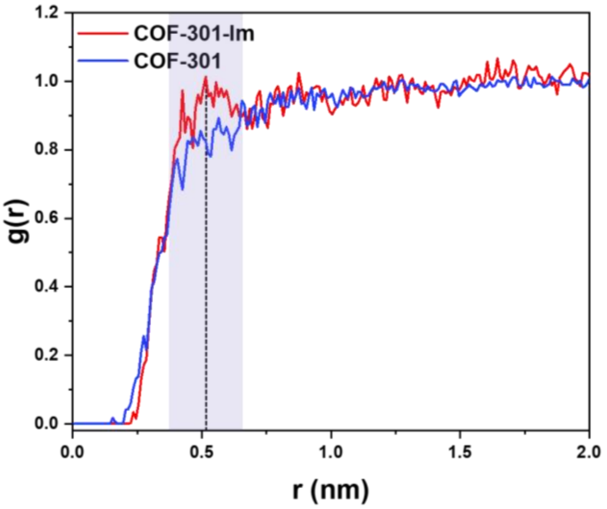

Figure S9. The radial distribution function images of PEGS molecular for COF-301 (blue) and COF-301-Im (red) fragments.

The spatial distribution function (SDF) indicates the probability distribution of PEGS molecules at varying distances from a given position within the framework fragment. Due to the electrostatic attraction between COF-301-Im and $PEGS^-$, the probability of $PEGS^-$ distribution at 0.5 nm is significantly higher for COF-301-Im compared to the COF-301 fragment. This clearly demonstrates the tighter binding between the COF-301-Im porous host and the PEGS guest. Furthermore, according to the fundamental principles of quantum mechanics, the interaction range of 0.5 nm falls within the scope of electrostatic forces, thereby confirming the objective existence of the corona-canopy electric double layer species formed by electrostatic interactions between COF-301-Im and $PEGS^-$.

CO_2 adsorption experiment and adsorption simulation of COF-301

CO_2 adsorption experiment

CO_2 high-pressure absorption measurements were acquired using a BSD-PH High Pressure Gas Sorption Analyzer. Approximately 150-300 mg of sample was loaded into a glass holder and evacuated to 5 mbar at 120 ºC to degas and dry the sample. The mass uptake (static capacity method) was then measured as a function of pressure up to a final pressure of 43 bar. Atmospheric (0-1 bar) CO_2 sorption isotherms for COF-301 and COF-301-Im were measured by using a Micrometrics ASAP 2020 instrument at 273 K.

Simulation of CO_2 adsorbtion

The Sorption module in Materials Studio 8.0 software was used, employing the COMPASS force field. The Ewald method and Atom-based method were used to handle long-range electrostatic interactions and van der Waals interactions, respectively. The fitting was performed at 298 K, first undergoing 5,000,000 steps of equilibration, followed by 10,000,000 steps of production.

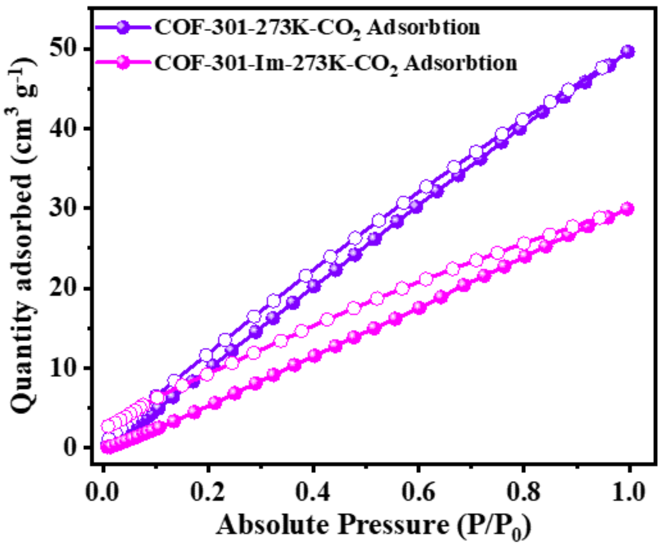

Figure S10. The CO_2 adsorption isotherms of COF-301 and COF-301-Im at 273K (0-1 bar).

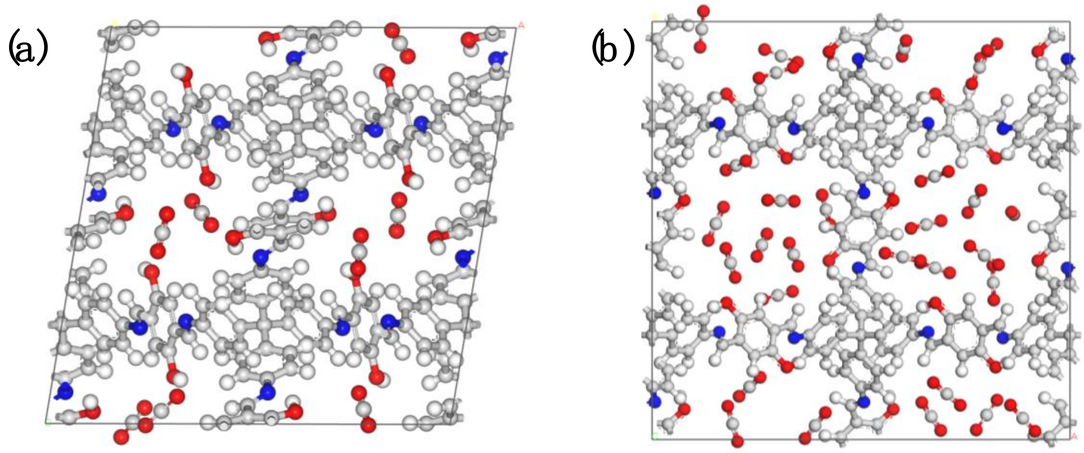

Figure S11. Schematic diagram of CO_2 adsorption by COF-301-***np*** phase (a) and COF-301-***lp*** phase (b) at 40 bar pressure.

Section 7. Porosity and Specific Surface Area Analysis.

Simulation of Connally surface and pore volumes

The N_2 adsorption experiments of COF-301 and COF-301-Im were carried out at the temperature of 77K with liquid nitrogen bath. The theoretical BET surface area and pore volume calculation results of COF-301-***np*** and COF-301-***lp*** are obtained by Materials Studio 8.0 Atoms Volumes & Surface tool, using N_2 as probe molecule.

$$\text{Pore Volume (cm}^3\text{/g)} = \frac{Cell\ Free\ Volume\ (\text{Å}3)}{Density\ (g/cm^3) \times Cell\ Volume\ (\text{Å}3)}$$

Table S5. The calculated pore volume of COF-301-***np*** and COF-301-***lp*** phases.

Entry	Cell Free Volume	Density	Cell Volume	Pore Volume
COF-301-***np***	778.8 $Å^3$	1.2082 cm^3/g	3522.44 $Å^3$	0.183 cm^3/g
COF-301-***lp***	2044.4 $Å^3$	0.7090 cm^3/g	5242.70 $Å^3$	0.550 cm^3/g

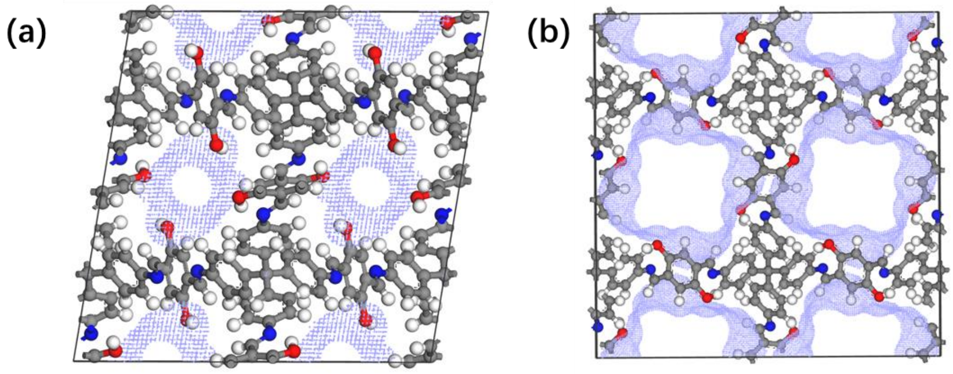

Figure S12. Visualization diagram of pore volume for COF-301-***np*** and COF-301-***lp***.

Positron annihilation lifetime spectroscopy (PALS)

When the material is exposed to a positron source (in this study, ^{22}NaCl), the positrons undergo thermalization, diffusion, and eventual annihilation upon being captured by electrons. The positron lifetime depends on the overlap of the positron wave function with the electron wave function in the material. Therefore, PALS can obtain the electron density distribution in the material by measuring the lifetime and intensity of positron annihilation sites, serving as an in-situ characterization technique for pores or pore volumes, particularly for determining the presence of cavity volumes. The positron lifetime is defined as the time interval between the injection of positrons into the material (indicated by the 1.274 MeV birth gamma ray) and the decay of the positron-electron pair (indicated by the two 0.511 MeV gamma rays traveling in opposite directions). In Figure 2b, the first lifetime (τ1) is attributed to the annihilation of positrons within the bulk of the materials (τ = 300–500 ps). The second lifetime (τ2) corresponds to ortho-positronium (*o-P*), a composite of a positron and an electron with parallel spins, forming in regions of low electron density such as voids, free volumes, interfaces, and cavities. The positron lifetime and intensity can be obtained through the fitting process. The *o-Ps* lifetime is typically related to the average radius ***r*** of the free volume units. According to the Tao-Eldrup model, assuming the free volume units are continuous spheres, the relationship between the radius r of the free volume and the *o-Ps* lifetime is as follows:

$$\tau_{o-Ps} = 0.5\left[1 - \frac{r}{r+\Delta r} + \frac{1}{2\pi} sin\left(\frac{2\pi r}{r+\Delta r}\right)\right]^{-1} (Equation\ 1)$$

Where Δr is the empirical electron layer thickness, which is taken to be 1.66 Å.

Section 8. Structure and performance characterization

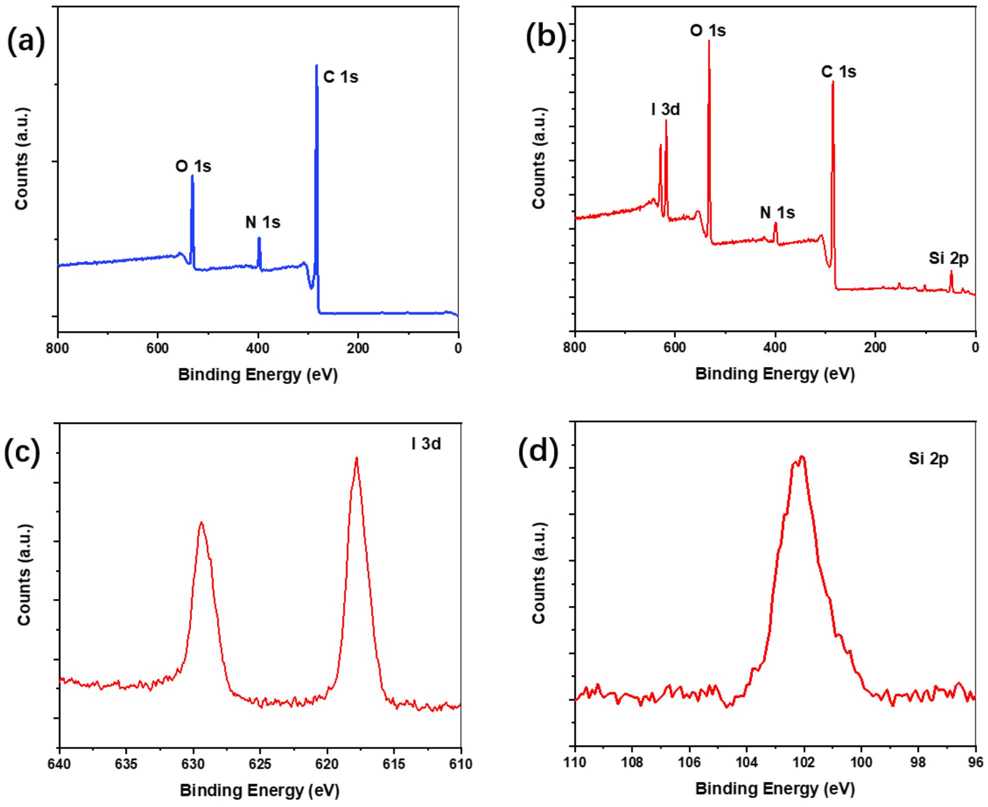

Figure S13. Survey XPS spectrum of COF-301 (a) and COF-301-Im (b). High-resolution I 3d (c) and Si 2p (d) XPS spectrum of COF-301-Im.

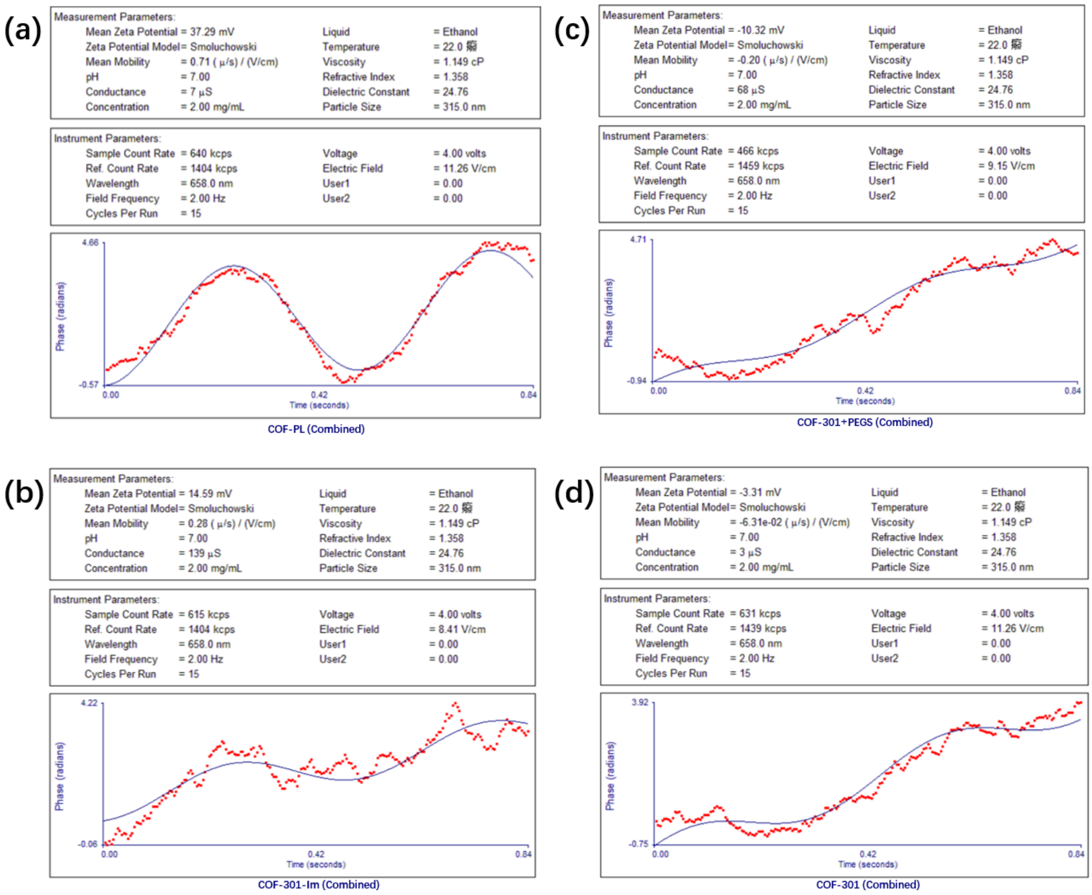

Figure S14. Zeta potentials of COF-301-PL (a), COF-301+PEGS (b), COF-301-Im (c) and COF-301 (d) ethanol dispersions.

Section 9. Details of catalytic cycloaddition of epoxide and carbon dioxide

In a typical experimental procedure, 3.0 g of COF-301-PL was placed in a high-pressure reactor at 298 K. After sealing, the reactor was purged with CO_2 three times to ensure complete removal of air from the system. Subsequently, the CO_2 pressure inside the reactor was increased to 20 bar and maintained until CO_2 adsorption reached saturation. The reactor was then opened to release the excess pressure, reducing the internal pressure to 1 bar. Finally, the reactor valve was quickly closed, and the reactor was rapidly cooled with liquid nitrogen for 3 minutes to stabilize the system. Afterward, the epichlorohydrin (1.56 mL, 20 mmol) and TBAB (32 mg, 0.1 mmol) were added to the autoclave. After being sealed again, the reaction was carried out at 120 ℃ for 10 h. After the reaction completed, small amounts of the mixture was withdrew, diluted with ethanol and filtered with a 0.22 μm membrane filter. The product was determined by gas chromatography. All the control experiments were carried out under the same reaction conditions with different carbon dioxide adsorbents.

Table S6. Cycloaddition of epichlorohydrin with CO_2

entry	Adsorbent	Yield(%)	Chloropropene Carbonate	Adsorbtion of CO_2
1	Blank	18.5	3.78 mmol	BLANK
2	3g PEGS	18.7	3.81 mmol	0.03 mmol
3	3g COF-301-PL	49.8	9.95 mmol	6.17 mmol
4	788 mg COF-301-Im	19.1	3.82 mmol	0.04 mmol
5	788 mg COF-301-Im + 2212 mg PEGS	28.9	5.78 mmol	1.96 mmol

For the COF-301-PL catalytic experiments involving the cycloaddition of epichlorohydrin under different CO_2 equilibrium pressures, only the equilibrium pressure of CO_2 was varied, while all other operational procedures remained identical to the previous process.

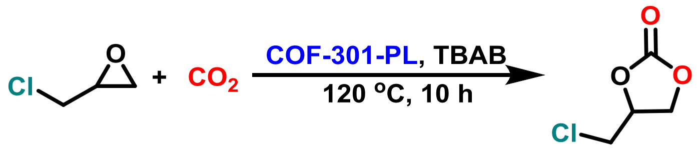

Table S7. Cycloaddition of epichlorohydrin with CO_2 at different pressures

P.S. The adsorbent used in all entries are 3g COF-301-PL.

Pressure (bar)	Blank CO_2 adsorption capacity	Yield(%)	Chloropropene Carbonate	Adsorbtion of CO_2
1	3.75 mmol	20.7	4.13 mmol	0.28 mmol
5	3.77 mmol	26.1	5.21 mmol	1.44 mmol
10	3.77 mmol	32.5	6.50 mmol	2.73 mmol
15	3.77 mmol	39.5	7.89 mmol	4.12 mmol
20	3.78 mmol	49.8	9.95 mmol	6.17 mmol
25	3.78 mmol	63.8	12.75 mmol	8.97 mmol

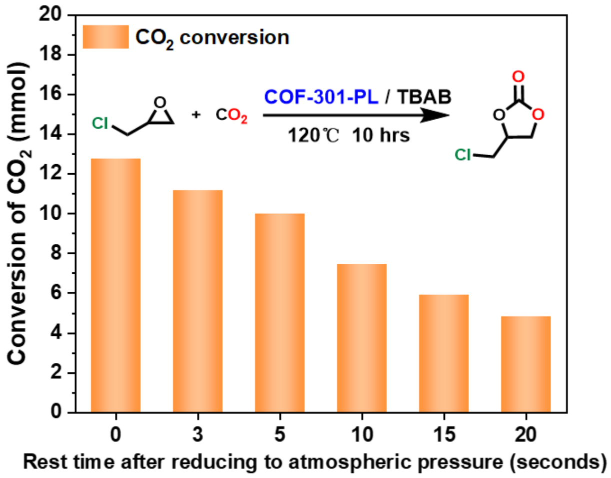

Figure S15. The yield of epichlorohydrin after different equilibrium times following COF-301-PL saturation under 25 bar of pressure.

To demonstrate the slow desorption of CO2 from COF-301-PL, the reactor containing COF-301-PL was first saturated with CO_2 under a pressure of 25 bar. Afterward, the CO_2 pressure was released, and the reactor pressure was restored to atmospheric pressure, marking the start of the timing. The remaining amount of CO_2 in the reaction system after different equilibration times was reflected by the yield of epichlorohydrin. (P.S. In this catalytic experiment, except for differences in CO_2 saturation pressure and equilibration time, all other operational methods and initial steps were exactly the same.)

Table S8. Yield of cycloaddition of epichlorohydrin after different equilibration times

Pressure (bar)	Equilibration time (seconds)	Chloropropene Carbonate
25	0	12.75 mmol
25	3	11.06 mmol
25	5	9.99 mmol
25	10	7.44 mmol
25	15	5.92 mmol
25	20	4.82 mmol

From the experimental results, it can be observed that the desorption of CO_2 in COF-301-PL does not occur immediately. Therefore, the CO_2 adsorption amount derived from the catalytic experiment with epichlorohydrin represents the minimum value of CO_2 adsorbed by the porous liquid. Nevertheless, this result still confirms that the pores of COF-301-PL undergo dynamic expansion under high pressure, leading to the storage of additional CO_2.

To evaluate the stability of COF-301-PL in catalytic experiments, we conducted cyclic catalytic performance tests. First, the reaction system after the catalytic process was ultrasonically washed three times with ethyl acetate to completely remove TBAB, epichlorohydrin, and chloropropylene carbonate. Subsequently, the washed COF-301-PL was subjected to rotary evaporation to thoroughly remove ethyl acetate, and the remaining liquid was collected. Finally, PEGS was replenished to restore COF-301-PL to its initial mass, and the cycling experiments were repeated three times following the original catalytic protocol.

Table S9. Yield of cycloaddition of epichlorohydrin after different cycle times.

P.S. The adsorbent used in all entries are 3g COF-301-PL.

Cycle times	Yield(%)	Chloropropene Carbonate	Adsorbtion of CO_2
1	49.8	9.95 mmol	6.17 mmol
2	48.5	9.70 mmol	5.92 mmol
3	48.7	9.73 mmol	5.95 mmol

The yield of propylene carbonate is calculated using the following formula:

Yield of propylene carbonate (%) = (amount of propylene carbonate / 20 mmol) × 100%

Amount of CO_2 additionally adsorbed = amount of propylene carbonate - the amount of CO_2 substance in the blank experiment

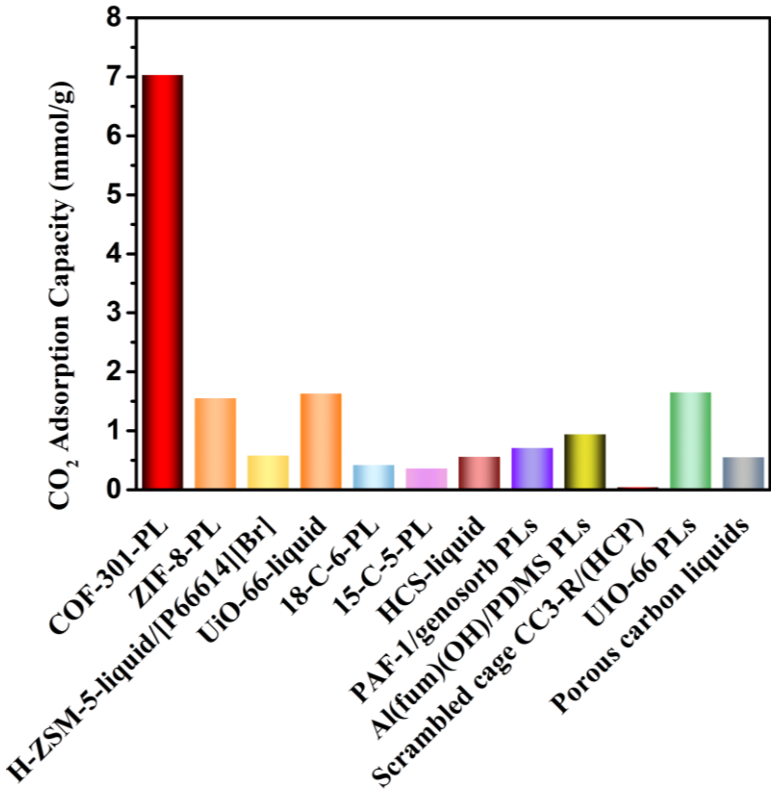

Figure S16. CO_2 adsorption performance of COF-301-PL and other reported PLs

Section 10. Reference

1. Grimme, S.; Antony, J.; Ehrlich, S.; Krieg, H., A Consistent and Accurate Ab Initio Parametrization of Density Functional Dispersion Correction (Dft-D) for the 94 Elements H-Pu. *J. Chem. Phys.* **2010,** *132* (15), 154104.
2. Grimme, S.; Ehrlich, S.; Goerigk, L., Effect of the Damping Function in Dispersion Corrected Density Functional Theory. *J. Comput. Chem.* **2011,** *32* (7), 1456-1465.
3. Humphrey, W.; Dalke, A.; Schulten, K., Vmd: Visual Molecular Dynamics. *J. Mol. Graphics Modell.* **1996,** *14* (1), 33-38.
4. Lu, T.; Chen, F. W., Multiwfn: A Multifunctional Wavefunction Analyzer. *J. Comput. Chem.* **2012,** *33* (5), 580-592.
5. Hess, B.; Kutzner, C.; van der Spoel, D.; Lindahl, E., Gromacs 4: Algorithms for Highly Efficient, Load-Balanced, and Scalable Molecular Simulation. *J. Chem. Theory Comput.* **2008,** *4* (3), 435-447.
6. Hub, J. S.; de Groot, B. L.; Grubmüller, H.; Groenhof, G., Quantifying Artifacts in Ewald Simulations of Inhomogeneous Systems with a Net Charge. *J. Chem. Theory Comput.* **2014,** *10* (1), 381-390.
7. Wang, J. M.; Wolf, R. M.; Caldwell, J. W.; Kollman, P. A.; Case, D. A., Development and Testing of a General Amber Force Field. *J. Comput. Chem.* **2004,** *25* (9), 1157-1174.
8. Bayly, C. I.; Cieplak, P.; Cornell, W. D.; Kollman, P. A., A Well-Behaved Electrostatic Potential Based Method Using Charge Restraints for Deriving Atomic Charges - the Resp Model. *J. Phys. Chem.* **1993,** *97* (40), 10269-10280.
9. O'Boyle, N. M.; Banck, M.; James, C. A.; Morley, C.; Vandermeersch, T.; Hutchison, G. R., Open Babel: An Open Chemical Toolbox. *J. Cheminformatics* **2011,** *3*, 33.
10. Brehm, M.; Thomas, M.; Gehrke, S.; Kirchner, B., Travis-a Free Analyzer for Trajectories from Molecular Simulation. *J. Chem. Phys.* **2020,** *152* (16), 164015.
11. Brehm, M.; Kirchner, B., Travis - a Free Analyzer and Visualizer for Monte Carlo and Molecular Dynamics Trajectories. *J. Chem. Inf. Model.* **2011,** *51* (8), 2007-2023.
